# ﻿Review of the genera and subgenera of the subtribe Aspilotina (Hymenoptera, Braconidae, Alysiinae), with a new illustrated key

**DOI:** 10.3897/zookeys.1229.142489

**Published:** 2025-02-26

**Authors:** Francisco Javier Peris-Felipo, Fernando Santa, Cornelis van Achterberg, Sergey A. Belokobylskij

**Affiliations:** 1 Bleichestrasse 15, CH–4058 Basel, Switzerland Unaffiliated Basel Switzerland; 2 Syngenta Crop Protection AG, Rosentalstrasse 67, CH–4058 Basel, Switzerland Syngenta Crop Protection AG Basel Switzerland; 3 Naturalis Biodiversity Center, 2333 CR Leiden, Netherlands Naturalis Biodiversity Center Leiden Netherlands; 4 Zoological Institute of the Russian Academy of Sciences, St Petersburg, 199034, Russia Zoological Institute of the Russian Academy of Sciences St Petersburg Russia

**Keywords:** Alysiini, *Aspilota* group, illustrated key, parasitoid of Diptera, Phoridae, re­descriptions

## Abstract

The genera and subgenera of the subtribe Aspilotina are reviewed. A new illustrated key to all accepted supraspecies taxa is provided. *Grandilota* Fischer, 2002, **stat. nov.** is proposed as subgenus of *Aspilota* Foerster, 1863. *Carinthilota* Fischer, 1975, **syn. nov.** is synonymised with *Alitha* Cameron, 1906, **stat. nov.** and the latter is treated as subgenus of *Dinotrema* Foerster, 1863; *Eudinostigma* Tobias, 1986, **syn. nov.** is considered a synonym of Dinotrema Foerster, 1863. Moreover, the new subgenus Pseudoprosapha**subgen. nov.** (type species: *Dinostigmastenosoma* van Achterberg, 1988) is described. Additionally, *Synaldotrema* Belokobylskij & Tobias, 2002, **stat. nov.** is treated as a separate genus. The following new combinations are proposed: Aspilota (Aspilota) ruficollis Stelfox & Graham, 1950, **comb. nov.**, Dinotrema (Alitha) lada (Belokobylskij, 1998), **comb. nov.**, D. (A.) longipennis (Cameron, 1906), **comb. nov.**, D. (A.) mavka (Belokobylskij, 1998), **comb. nov.**, D. (A.) parapsidalis (Fischer, 1975), **comb. nov.**, D. (A.) vechti (van Achterberg, 1988), **comb. nov.**, Dinotrema (Dinotrema) alox (van Achterberg, 1988), **comb. nov.**, D. (D.) entabeniense (Fischer, 2009), **comb. nov.**, D. (D.) latum (Chen & Wu, 1994), **comb. nov.**, D. (D.) planiceps (Fischer, Tormos & Pardo, 2006), **comb. nov.**, D. (D.) subpulvinatum (Fischer, 2009), **comb. nov.**, D. (Pseudoprosapha) stenosoma (van Achterberg, 1988), **comb. nov.**, D. (Synaldis) bienesae (Fischer, Tormos & Pardo, 2006), **comb. nov.**, D. (S.) fischeri (Tobias, 1986), **comb. nov.**, D. (S.) latistigma (Fischer, 1962), **comb. nov**., D. (S.) planiceps (Fischer, Tormos & Pardo, 2006), **comb. nov.**, D. (Synaldis) cespitator (Belokobylskij, 2004), **comb. nov.**, D. (S.) perfidum (Fischer, 1970), **comb. nov.**, D. (S.) trematosum (Fischer, 1967), **comb. nov.** and *Paneremafulvicornis* (Haliday, 1838), **comb. nov.**

## ﻿Introduction

The parasitoid wasps of the subtribe Aspilotina Belokobyskij & Tobias, 2002 are the largest aggregation within the braconid tribe Alysiini (Hymenoptera: Braconidae: Alysiinae) with approximately 850 valid species worldwide (Yu et al. 2016). Specimens of this group are nearly always small, with a body length of 1.0–3.0 mm, and the body colour is predominantly dark brown to black.

Despite host-parasitoid relationships in the Alysiini are yet insufficiently known, already 29 families of cyclorrhaphous Diptera have been listed in the literature as their hosts. Most of them belong to the families Agromyzidae, Anthomyiidae, Calliphoridae, Drosophilidae, Muscidae, Phoridae, Sarcophagidae, and Tephritidae (Yu et al. 2016; Kostromina et al. 2016; Peris-Felipo and Belokobylskij 2018a), whose biological preferences vary from phytophagous to saprophagous and necrophagous. The members of the subtribe Aspilotina predominantly develop into larvae of the dipteran family Phoridae or humpbacked flies, which mainly feed on decaying organic matter (van Achterberg 1988). Other host records (e.g., of Agromyzidae and Drosophilidae) are mostly old and need reconfirmation, since Phoridae also consume insects from decaying organic matter.

Over the years, the genera included in this subtribe have changed statuses. For example, van Achterberg (1988) included the type species of *Synaldis* Foerster, 1863 in the genus *Dinotrema* Foerster, 1863. However, later publications by Fischer (1993a, 1993b), Belokobylskij (2002, 2004a, 2004b), Tobias (2003a, 2003b, 2004, 2006), and Peris-Felipo (Peris-Felipo et al. 2014a, 2014b; Peris-Felipo and Belokobylskij 2017) continued to consider *Synaldis* as a taxonomically valid genus separated from *Dinotrema* due to rather a stable diagnostic character, the complete absence of the vein 2-SR in fore wing and with the vein r not angled with vein 3-SR, resulting in a gently curved or straight vein. Groups based on the reduction of this vein (which is a wide-spread phenomenon in the subtribe Aspilotina) are likely derived lineages within a genus, and there is even no proof that all species included in e.g., *Synaldis* sensu stricto are belonging to the same lineage. Recently, Zhu et al. (2017) included Synaldis sensu stricto as subgenus of Dinotrema and synonymised *Adelphenaldis* Fischer, 2003 and *Regetus* Papp, 1999 with *Eusynaldis* Zaykov & Fischer, 1982, including the latter as a subgenus in *Aspilota* Foerster, 1863. In this paper, we review the generic status of the taxa within the subtribe Aspilotina and compile a new illustrated identification key to all accepted genera and subgenera.

## ﻿Materials and methods

The revisions of the type species of the *Aspilota* group genera (= subtribe Aspilotina) carried out during many years and checking a large number of *Aspilota*, *Dinotrema*, and *Orthostigma* species allows us to recognise the main diagnosis characters to classify the almost 850 valid species worldwide. These characters are: paraclypeal fovea remained removed from or reaching the border of the eye; mandible without or sometimes with transverse carina; furrow between antennal socket and eye absent or present; notauli mainly absent in dorsal view or sometimes well developed; scutellum without or with transverse crenulated depression; position of the origin of the vein r; pterostigma almost not differentiated, very narrow, or sometimes well isolated and wide; vein 2-SR of fore wing present or absent; subbasal cell of hind wing distally closed or open; first subdiscal cell of fore wing distally close or open; main cells of the hind wing closed or open; fore femur narrow and simple or wide and with tooth; first metasomal tergite with or without dorsope; second metasomal tergite entirely smooth or rarely sculptured basally; hypopygium not retracted under distal metasomal segments or sometimes strongly retracted.

For the terminology of morphological features, sculpture, and measurements (including for mandibles) see Peris-Felipo et al. (2014a); for wing venation nomenclature see van Achterberg (1993); for measurements of the marginal cell see Peris-Felipo and Belokobylskij (2017). The material was imaged using a Digital Keyence® VHX-2000 and Adobe Photoshop® imaging system.

The specimens examined are preserved in the entomological collections at the institutions listed below:

**ANIC**Australian National Insect Collection (Canberra, Australia)

**BMNH** The Natural History Museum (London, U.K.)

**ENV** Entomological Collection of the University of Valencia (Valencia, Spain)

**HNHM**Hungarian Natural History Museum (Budapest, Hungary)

**MNHN**Museum National d’Histoire Naturelle (Paris, France)

**NHMW**Naturhistorisches Museum (Vienna, Austria)

**NHMB** Museum für Naturkunde (Berlin, Germany)

**NHMD** Natural History Museum of Denmark (Copenhagen, Denmark)

**NMA** Naturhistorisk Museum Aarhus (Aarhus, Denmark)

**NMNH**Smithsonian National Museum of Natural History (Washington, U.S.A.)

**PFEC** F.J. Peris-Felipo Private Entomological Collection (Basel, Switzerland)

**QMBA**Queensland Museum (Brisbane, Australia)

**RMNH**Naturalis Biodiversity Center (Leiden, The Netherlands)

**ZISP**Zoological Institute of the Russian Academy of Sciences (St Petersburg, Russia)

**ZSSM**Zoologische Staatssammlung Münchenn (München, Germany).

To establish the position and relationship between genera and subgenera, a multivariate statistical approach was used to build the cladogram (Table 1). Specifically, as the list of characters, plesiomorphic and apomorphic states, are attributes or qualitative variables, we performed a multiple correspondence analysis (MCA) (Greenacre 2017) to outline the relationship between the 17 characters. MCA builds a new set of latent variables (scores) which summarises the contained information in the variables and represent them in a geometric space. The analysis was conducted using R v. 4.4.1, with the FactoMineR (v. 2.11) and factoextra (v. 1.0.7) packages for MCA implementation and visualisation. We selected 10 scores, which accounted for at least 90% of the retained variability, effectively reducing the dimensionality from the original 17 plesiomorphic attributes to ten summary scores. Subsequently, we performed hierarchical clustering on these selected scores using Ward’s procedure (Husson 2017), implemented through the cluster package (v. 2.1.6) in R. This clustering approach allowed us to identify groups of genera that share common characters. The combination of MCA and hierarchical clustering provided a robust framework for constructing the cladogram based on the shared attributes among the studied genera.

**Table 1.** The characters used for the cladogram construction.

Character	Plesiomorphic (0)	Apomorphic (1)
Furrow between antennal socket and eye	absent	present
Paraclypeal fovea	small to medium-sized	large and reaching eye border
Mandible	without transverse carina	with distinct submedial transverse carina
First flagellar segment	longer than second segment	equal to or shorter than second segment
Notauli	complete	posterior half absent
Scutellum	without depression posteriorly	with transverse depression posteriorly
Pterostigma	very slender	secondary widened
Vein r of fore wing originated	from the basal quarter of pterostigma	far from the base of pterostigma, arising near its middle
Subbasal cell of hind wing distally	closed	open
First subdiscal cell of fore wing distally	closed	open
Vein 2-SR of fore wing	present	absent
Hind wing cells	closed	open
Fore femur	simple without tooth	with wide ventral tooth
First tergite of metasoma	without dorsope	with dorsope
Hypopygium of metasoma	not retracted anteriorly under posterior tergites	distinctly retracted anteriorly under posterior tergites
Second tergite	smooth	sculptured
Clypeus	not protruding	protruding

## ﻿Taxonomic part

**Class Hexapoda Blainville, 1816**

**Order Hymenoptera Linnaeus, 1758**

**Family Braconidae Nees, 1811**

**Subfamily Alysiinae Leach, 1815**

**Tribe Alysiini Leach, 1815**

**Subtribe Aspilotina Belokobylskij & Tobias, 2002**

**Figure 1.**
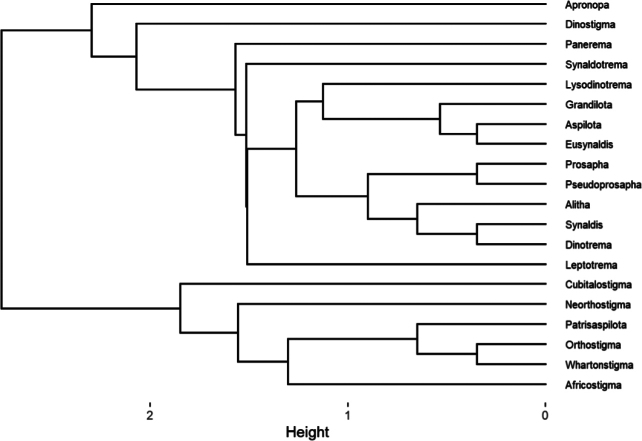
Subtribe Aspilotina cladogram based on the diagnostic characters from Table 1.

The relation between the diagnostic characters has allowed the construction of a cladogram of the relationships in the Aspilotina (Fig. 1) in whose two main different linages are clearly distinguished from the beginning: *Aspilota* group and *Orthostigma* group. The *Aspilota* group linage consists of eight genera: *Apronopa* van Achterberg, 1980, *Aspilota* Foerster, 1863, *Dinostigma* Fischer, 1966, *Dinotrema* Foerster, 1863, *Leptotrema* van Achterberg, 1988, *Lysodinotrema* Fischer, 1995, *Panerema* Foerster, 1863, and *Synaldotrema* Belokobylskij & Tobias, 2002, stat. nov. Moreover, the genus *Aspilota* is formed by the subgenera *Aspilota* Foerster, 1863, sensu stricto, *Eusynaldis* Zaykov & Fischer, 1982, and *Grandilota* Fischer, 2002, stat. nov., while the genus *Dinotrema* contains the subgenera *Alitha* Cameron, 1906, stat. nov., *Dinotrema* Foerster, 1863 (sensu stricto), *Prosapha* Foerster, 1863, Pseudoprosapha subgen. nov., and *Synaldis* Foerster, 1863.

*Orthostigma* group contains three genera: *Cubitalostigma* Fischer, 1998, *Neorthostigma* Belokobylskij, 1998, and *Orthostigma* Ratzeburg, 1844. The latter is made up of four subgenera: *Africostigma* Fischer, 1995, *Orthostigma* Ratzeburg, 1844, sensu stricto, *Patrisaspilota* Fischer, 1995, and *Whartonstigma* Peris-Felipo, 2020. The distances between genera/subgenera generated from the multivariate statistical approach of the diagnostic characters are provided in Appendix 1.

### ﻿Synopsis of the genera and subgenera of the subtribe Aspilotina

***Aspilota* group**

Genus *Apronopa* van Achterberg, 1980

Genus *Aspilota* Foerster, 1863

subgenus Aspilota Foerster, 1863, sensu stricto

subgenus Eusynaldis Zaykov & Fischer, 1982 (= *Regetus* Papp, 1999; *Adelphenaldis* Fischer, 2003)

subgenus Grandilota Fischer, 2002, stat. nov.

Genus *Dinostigma* Fischer, 1966

Genus *Dinotrema* Foerster, 1863 (Syn.: *Pterusa* Fischer, 1958; *Eudinostigma* Tobias, 1986, syn. nov.)

subgenus Alitha Cameron, 1906, stat. nov. (= *Carinthilota* Fischer, 1975, syn. nov.)

subgenus Dinotrema Foerster, 1863, sensu stricto

subgenus Prosapha Foerster, 1863

subgenus Pseudoprosapha subgen. nov.

subgenus Synaldis Foerster, 1863

Genus *Leptotrema* van Achterberg, 1988

Genus *Lysodinotrema* Fischer, 1995

Genus *Panerema* Foerster, 1863

Genus *Synaldotrema* Belokobylskij & Tobias, 2002, stat. nov.

***Orthostigma* group**

Genus *Cubitalostigma* Fischer, 1998

Genus *Neorthostigma* Belokobylskij, 1998

Genus *Orthostigma* Ratzeburg, 1844

subgenus Africostigma Fischer, 1995

subgenus Orthostigma Ratzeburg, 1844, sensu stricto

subgenus Patrisaspilota Fischer, 1995

subgenus Whartonstigma Peris-Felipo, 2020

#### ﻿Subtribe Aspilotina Belokobyskij & Tobias, 2002

Aspilotina Belokobyskij and Tobias 2002: 2.

#### ﻿*Aspilota* group

**Morphological diagnosis.** See van Achterberg (1988).

##### ﻿Genus Apronopa

Taxon classificationAnimaliaHymenopteraBraconidae

van Achterberg, 1980

9431B81B-96F0-5CBF-8F36-DBF3697BFDE9

Apronopa van Achterberg, 1980: 75; Tobias 1986: 195; Fischer 1991: 8; Wharton 1994: 640; Belokobylskij 1998a: 169, 217; Belokobylskij and Tobias 2007: 10; Yu et al. 2016; Peris-Felipo and Belokobylskij 2018b: 144.

###### Type species.

**Figure 2.**
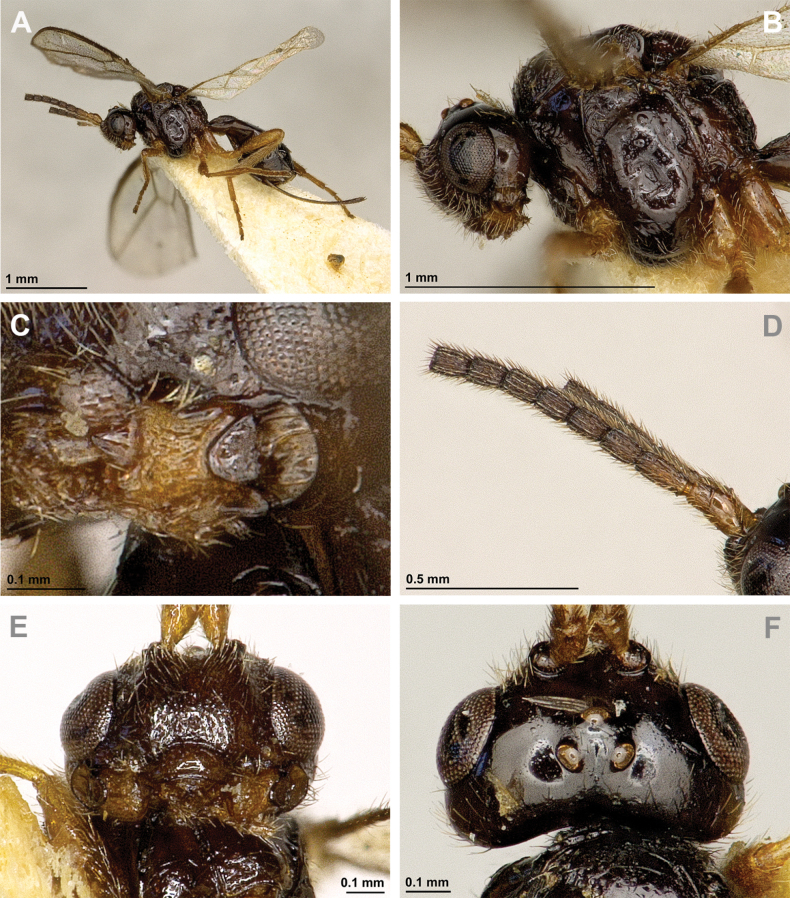
*Apronopahaeselbarthi* van Achterberg, 1980 (holotype, female) **A** habitus, lateral view **B** head and mesosoma, lateral view **C** mandible **D** antenna **E** head, front view **F** head, dorsal view.

**Figure 3.**
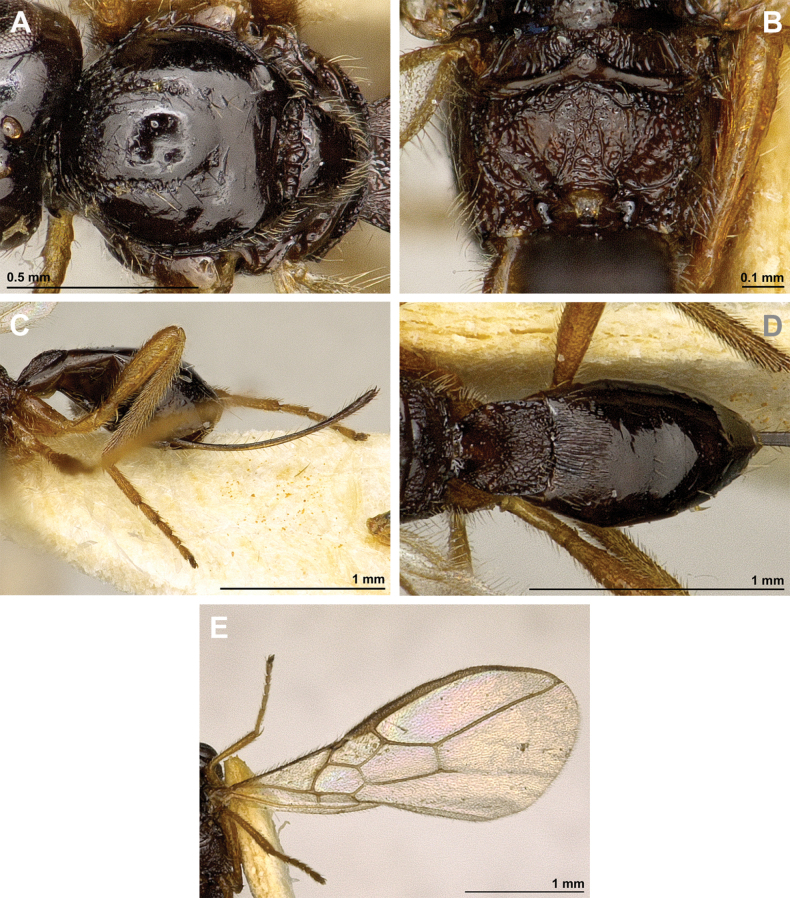
*Apronopahaeselbarthi* van Achterberg, 1980 (holotype, female) **A** mesonotum, dorsal view **B** propodeum **C** legs, metasoma and ovipositor, lateral view **D** metasomal tergites, dorsal view **D** fore wing.

*Apronopahaeselbarthi* van Achterberg, 1980, by original designation (Figs 2, 3).

###### Material examined.

***Holotype*** (*Apronopahaeselbarthi*) Germany: • ♀, Dransfeld, B/L 2.vi.1966 (Haeselbarth leg.) (ZSSM). ***Paratypes*** (*Apronopahaeselbarthi*) Germany: • 1 ♀, 1 ♂, Schotten, Hessen, Fi., Streu, v.1967 (Haeselbarth leg.) (♀ in RMNH, ♂ in ZSSM).

###### Diagnosis.

Mandible small, simple, robust, tridentate. Paraclypeal fovea short, remaining far from inner margin of eyes. Mesoscutum without medio-posterior pit; notauli absent in posterior half of mesoscutum; precoxal sulcus always present; propodeum smooth or with different types of sculpture and sometimes with longitudinal or transverse carinae. Marginal cell of fore wing never shortened; vein r originating approximately from basal quarter of pterostigma; vein 2-SR always present and distinctly sclerotized; veins m-cu and cu-a distinctly postfurcal; first subdiscal cell always closed postero-apically by CU1a vein. Metasoma of ♀ more or less distinctly compressed laterally. First metasomal tergite without dorsope; second tergite often longitudinally striate medially. Ovipositor sheath not longer than metasoma.

###### Remarks.

This is a small genus with only three described species exclusively from the Palaearctic region (two of these species have an East Palaearctic distribution). Unfortunately, there is no data about its biology. *Apronopa* is characterised by three distinct diagnostic characters (van Achterberg 1980; Peris-Felipo and Belokobylskij 2018b): the dorsope of the first metasomal tergite are absent, the ovipositor has a distinct dorsal nodus subapically and the second metasomal tergite is sculptured basally (except in *A.levis* Papp, 2007). The combination of these features is unknown in other Aspilotina and supports well the separate generic status of this taxon.

##### ﻿Genus Aspilota

Taxon classificationAnimaliaHymenopteraBraconidae

Foerster, 1863

3A5B8B7C-F6B2-50C2-9127-4F48F518B8BE

**Figure 4.**
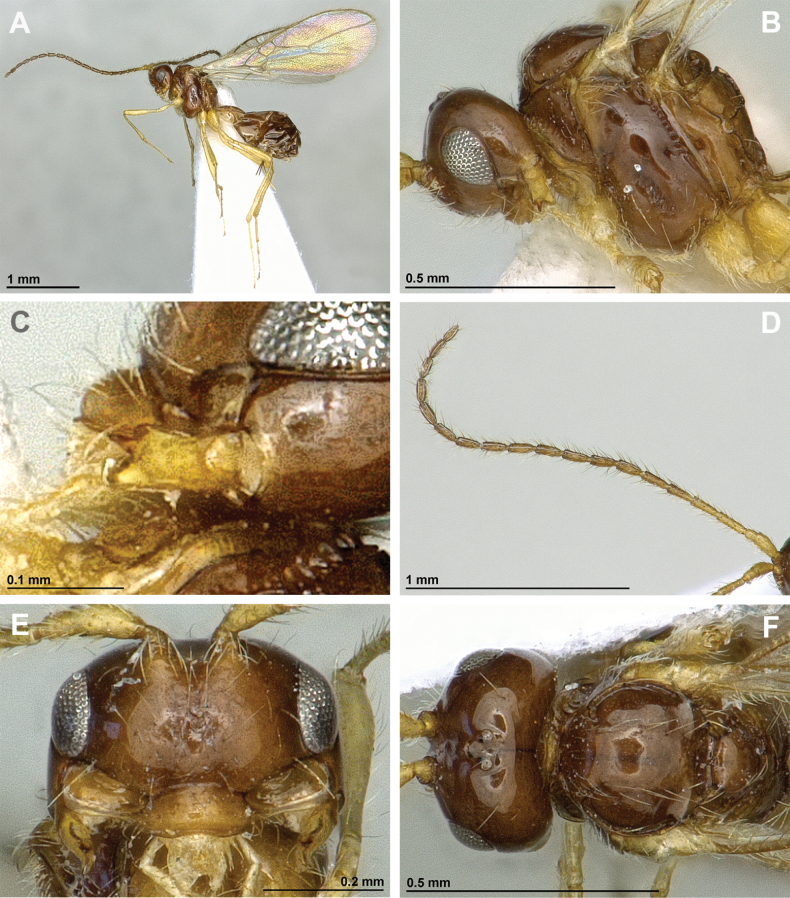
Aspilota (Aspilota) ajara Peris-Felipo, 2016 (holotype, female) **A** habitus, lateral view **B** head and mesosoma, lateral view **C** mandible **D** antenna **E** head, front view **F** head and mesonotum, dorsal view.

**Figure 5.**
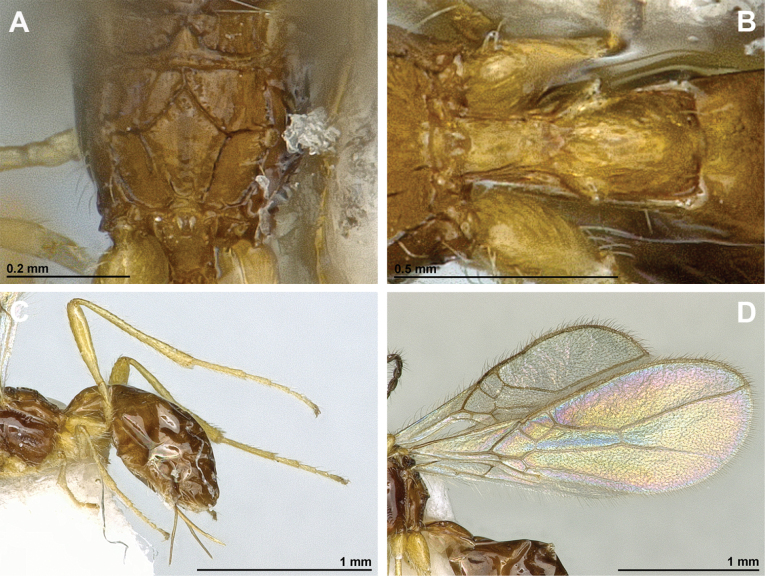
Aspilota (Aspilota) ajara Peris-Felipo, 2016 (holotype, female) **A** propodeum **B** first metasomal tergite, dorsal view **C** legs, metasoma and ovipositor, lateral view **D** fore and hind wings.

**Figure 6.**
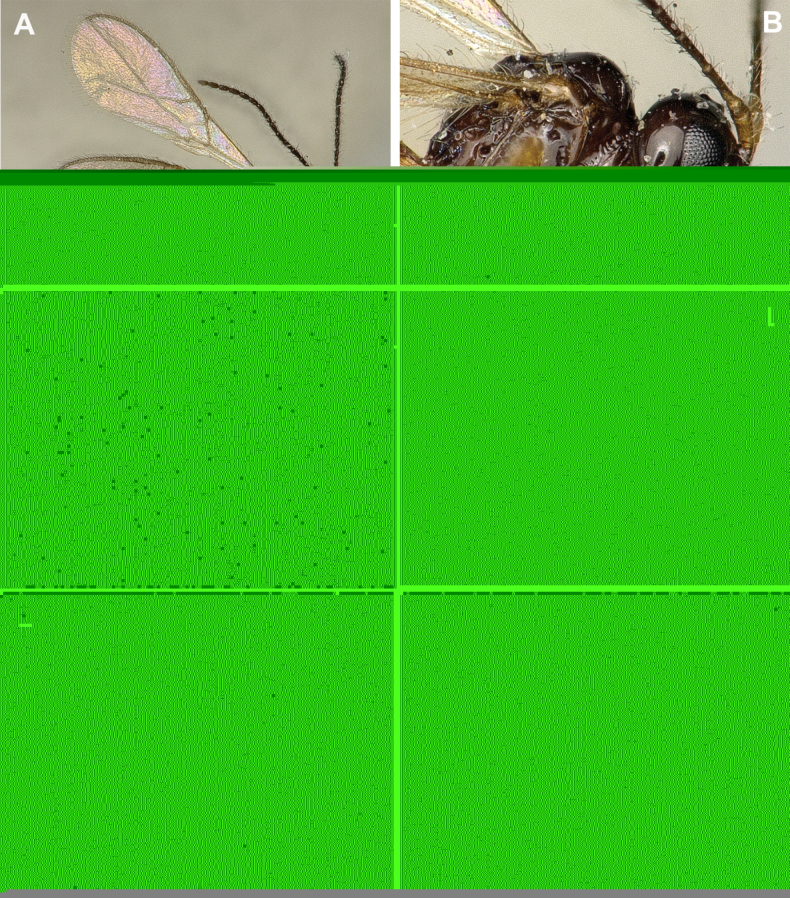
Aspilota (Eusynaldis) varinervis (Zaykov & Fischer, 1972) (holotype, male) **A** habitus, lateral view **B** head and mesosoma, lateral view **C** mandible **D** antenna **E** head, front view **F** head and mesonotum, dorsal view.

**Figure 7.**
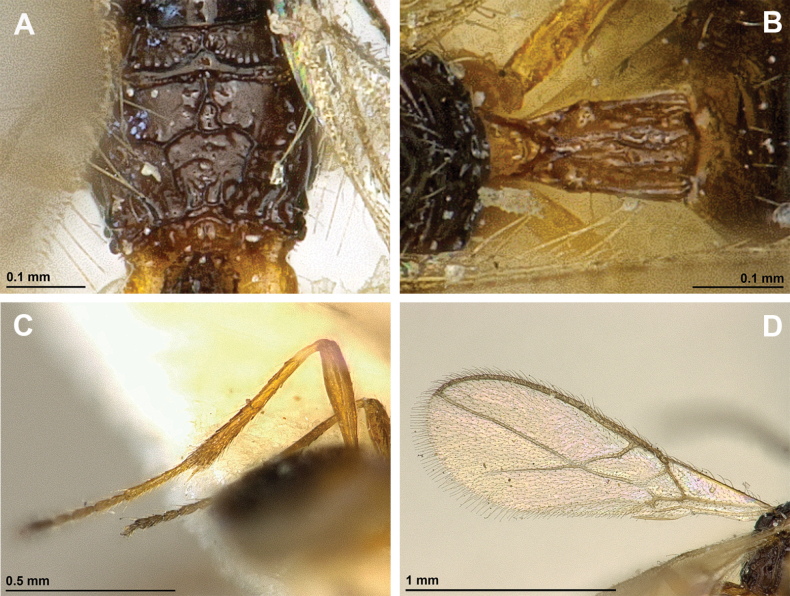
Aspilota (Eusynaldis) varinervis (Zaykov & Fischer, 1972) (holotype, male) **A** propodeum **B** first metasomal tergite, dorsal view **C** hind leg, lateral view **D** fore wing.

**Figure 8.**
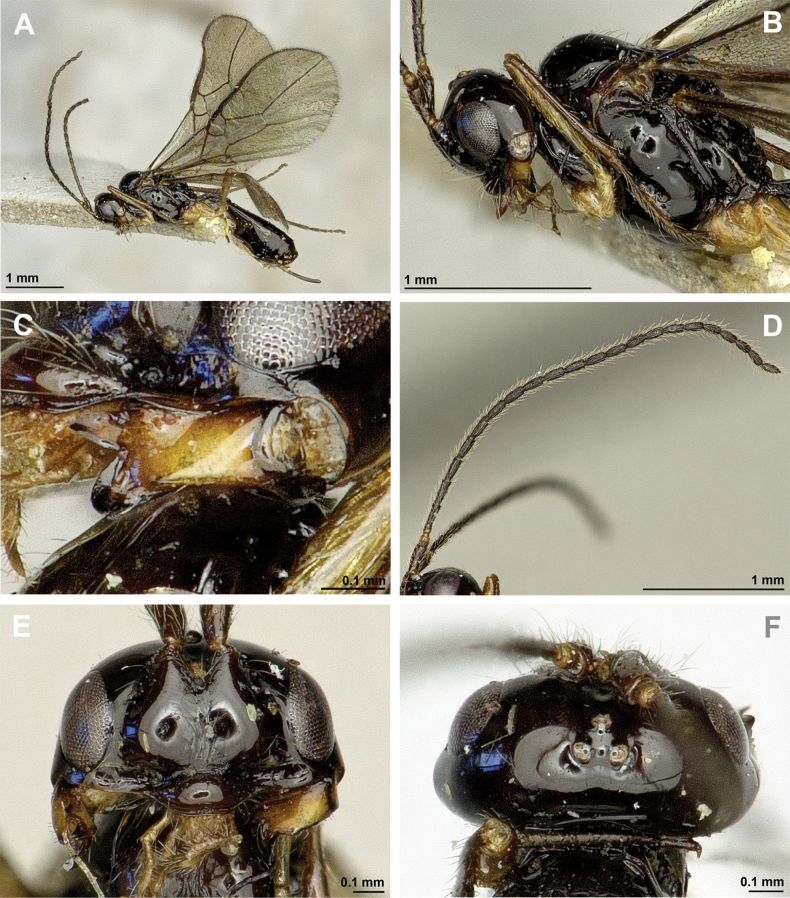
Aspilota (Grandilota) kenyaensis (Fischer, 2002), comb. nov. (holotype, female) **A** habitus, lateral view **B** head and mesosoma, lateral view **C** mandible **D** antenna **E** head, front view **F** head, dorsal view.

**Figure 9.**
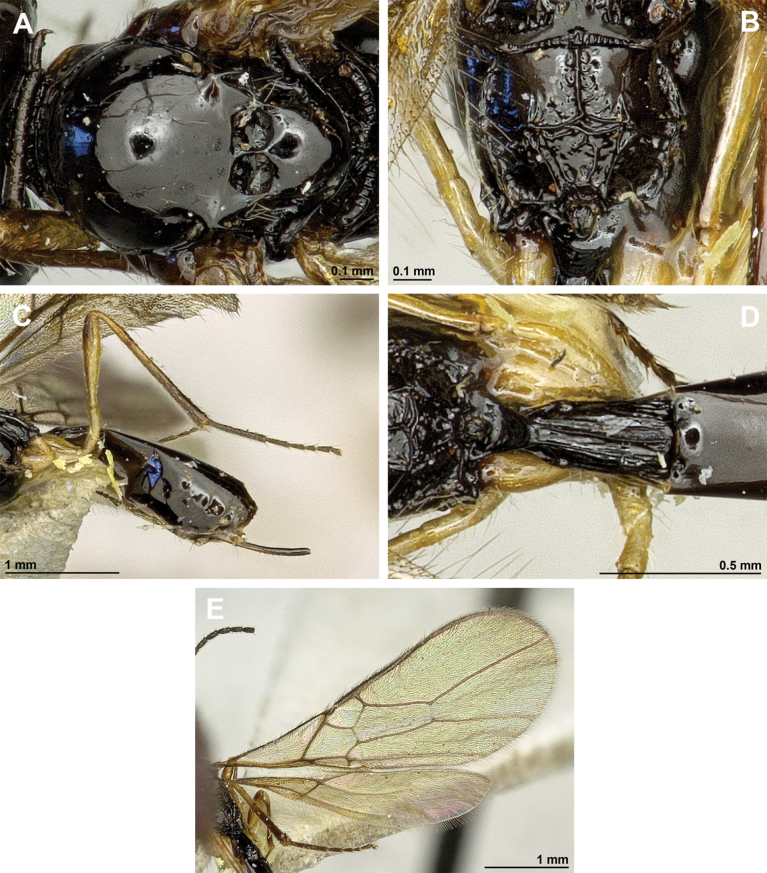
Aspilota (Grandilota) kenyaensis (Fischer, 2002), comb. nov. (holotype, female) **A** mesonotum, dorsal view **B** propodeum. **C** legs, metasoma and ovipositor, lateral view **D** first metasomal tergite, dorsal view **E** fore and hind wings.

Figs 4 , 5 , 6 , 7 , 8 , 9

Aspilota Foerster, 1863: 268; Shenefelt 1974: 966; Wharton 1980: 84; van Achterberg 1988: 9; Chen and Wu 1994: 49; Belokobylskij 1998a: 218; Wharton 2002: 34; Yu et al. 2016.
Dipiesta Foerster, 1863: 268 (synonymised with Aspilota Foerster by Szépligeti (1904)).
Eusynaldis Zaykov & Fischer, 1982: 70; van Achterberg 1988: 9 (as synonym of Aspilota Foerster); Zhu et al. 2017: 19; Peris-Felipo and Belokobylskij 2019: 21.
Regetus Papp, 1999: 391; Fischer 2002: 101; Zhu et al. 2017: 19 (as synonym of Aspilota Foerster).
Grandilota Fischer, 2002: 103; Yu et al. 2016.
Adelphenaldis Fischer, 2003: 41; Peris-Felipo et al. 2012: 287; 2014b: 571; Yu et al. 2016; Zhu et al. 2017: 19 (as synonym of Aspilota Foerster).

###### Type species.

*Alysiaruficornis* Nees von Esenbeck, 1834, by monotypy.

###### Diagnosis.

Mandible small, simple, tridentate, often with upper (first) tooth diminished with respect to lower (third) tooth. Paraclypeal fovea large, reaching inner margin of eyes. Mesoscutum with or without medio-posterior pit; notauli present only in anterior part of mesoscutum; precoxal sulcus almost always present; propodeum smooth or more common with different types of sculpture and sometimes with longitudinal and/or transverse carinae, rarely forming areas. In fore wing, marginal cell never shortened; vein r originating from basal quarter of pterostigma; vein 2-SR often present and usually distinctly sclerotised but absent in subgenus Eusynaldis; veins m-cu and cu-a postfurcal; first subdiscal cell always closed postero-apically by vein CU1a. In hind wing, subbasal cell usually closed. Metasoma of ♀ more or less distinctly compressed laterally; second tergite always smooth. Ovipositor sheath usually not longer than metasoma.

###### Remarks.

Members of the genus *Aspilota* are frequently encountered as they are one of the most common genera among Alysiini wasps. It is mainly distributed in forested and humid areas of the Holarctic region and only a few species have been already recorded from other zoogeographic regions. This genus is undersampled in the tropics where their main hosts (Phoridae) have the greatest diversity.

*Aspilota* species are koinobiont endoparasitoids of larvae, mainly of the family Phoridae (Diptera). Previous reports established *Aspilota* as parasitoid of the families Anthomyiidae, Lonchaeidae, Muscidae, Platypezidae, Sarcophagidae, Syrphidae, and Tephritidae. However, these hosts need to be especially reconfirmed. The records of lepidopterous and hymenopterous larvae as hosts (families Erebidae, Bucculatricidae, Lasiocampidae, and Tortricidae, and family Tenthredinidae, respectively) are extremely doubtful because the known biology and perhaps were concerned to the Phoridae living in dead larvae of the species from these families.

The genus *Aspilota* contains three subgenera, *Aspilota* sensu stricto, *Eusynaldis* Zaykov & Fischer, 1982, and *Grandilota* Fischer, 2002.

##### ﻿Subgenus Aspilota

Taxon classificationAnimaliaHymenopteraBraconidae

Foerster, 1863, sensu stricto

A16B411B-7FDB-5B4D-86FA-5FDEE0A75B1B

Figs 4 , 5

Aspilota Foerster, 1863: 268; Shenefelt 1974: 966; Wharton 1980: 84; van Achterberg 1988: 9; Chen and Wu 1994: 49; Belokobylskij 1998a: 218; Wharton 2002: 34; Yu et al. 2016.
Dipiesta Foerster, 1863: 265.

###### Type species.

*Alysiaruficornis* Nees von Esenbeck, 1834, by monotypy.

###### Material examined.

Numerous species from the Palaearctic, Nearctic, and Neotropical regions were reviewed (e.g., Peris-Felipo and Belokobylskj 2014; Peris-Felipo et al. 2016a, 2016b, 2016c, 2016d).

###### Remarks.

This largest and easily recognised subgenus includes most of *Aspilota* species. *Pterusaruficollis* (Stelfox & Graham, 1950) is returned to *Aspilota* as *A.ruficollis* Stelfox & Graham, 1950, comb. nov. after the revision of type because the paraclypeal fovea are wide and reaching the inner margin of the eyes. Its new generic position is also supported by Fischer’s re-description of the species (Fischer 1972: 436).

##### ﻿ Subgenus Eusynaldis

Taxon classificationAnimaliaHymenopteraBraconidae

Zaykov & Fischer, 1982

15FB199F-3E49-5A42-8A9E-7C0D4801C623

Eusynaldis Zaykov & Fischer, 1982: 70; Zhu et al. 2017: 19 (as subgenus); Peris-Felipo and Belokobylskij 2019: 21.
Regetus Papp, 1999: 391; Fischer 2002: 101; Zhu et al. 2017: 19 (as synonym).
Adelphenaldis Fischer, 2003: 41; Peris-Felipo et al. 2012: 287; 2014b: 571; Yu et al. 2016; Zhu et al. 2017: 19 (as synonym).

###### Type species.

*Eusynaldisvarinervis* Zaykov & Fischer, 1972, by monotypy (Figs 6, 7).

###### Material examined.

***Holotype****Regetusbalcanicus* [= Aspilota (Eusynaldis) globipes] Former Yugoslavia: • ♀, Kosovo, Mts. Sar, Brezovica, 900–1200 m, 20–23.v.1971 (Papp & Hortatovich leg.) (HNHM) [Hym. Typ. No. Mus. Budapest 7878].

###### Diagnosis.

Subgenus Eusynaldis shares all diagnostic characters of *Aspilota* sensu stricto, except the absent vein 2-SR of the fore wing.

###### Remarks.

*Regetus* Papp, 1999 and *Adelphenaldis* Fischer, 2003 are junior synonyms of *Eusynaldis* Zaykov & Fischer, 1982 because both taxa are characterised by the same diagnostic features (Zhu et al. 2017). Moreover, the study of the holotype of *Regetusbalcanicus* Papp, 1999 (the type species of *Regetus*) showed this species to be a junior synonym of Aspilota (Eusynaldis) globipes (Fischer, 1962) (Peris-Felipo and Belokobylskij 2019).

##### ﻿ Subgenus Grandilota

Taxon classificationAnimaliaHymenopteraBraconidae

Fischer, 2002, stat. nov.

CE9EBC54-8E67-518F-ABD9-00A256AA8595

Grandilota Fischer, 2002: 103; Yu et al. 2016.

###### Type species.

*Grandilotakenyaensis* Fischer, 2002, by original designation (Figs 8, 9).

###### Material examined.

***Holotype*** (*Grandilotakenyaensis*). Kenya: • ♀, Mt. Elgon Natural Park, bamboo (Arundinaria alpine) thicket, 2740 m; swept. No. 496, 22.i.1992 (G. Varkonyl leg.) [Hym. Typ. No. Mus. Budapest 11673] (HNHM). ***Paratype*** (*Grandilotakenyaensis*) Kenya: • ♀, same label as holotype but [Hym. Typ. No. Mus. Budapest 11674] (HNHM).

###### Diagnosis.

Mandible well developed, tridentate, with upper (first) tooth diminished to respect to lower (third) tooth. Paraclypeal fovea long, reaching inner margin of eyes. Mesoscutum without mesoscutal pit; notauli present only in anterior half of mesoscutum; precoxal sulcus present; propodeum with pentagonal areola, delineated by carinae. In fore wing, marginal cell reaching apex of wing; vein r originating from basal quarter of pterostigma; vein 2-SR present and sclerotised; veins m-cu and cu-a postfurcal; first subdiscal cell closed postero-apically by CU1a vein. In hind wing, subbasal cell open. Metasoma of ♀ more or less distinctly compressed laterally. Ovipositor sheath not longer than metasoma.

###### Remarks.

This subgenus has only one known species, Aspilota (Grandilota) kenyaensis Fischer, 2002, from Kenya and shares the main characters with *Aspilota* sensu stricto, however, the subbasal cell of the hind wing open distally (absent vein cu-a) and the wing membrane is pigmented, which distinguishes it at the subgeneric level.

##### ﻿Genus Dinostigma

Taxon classificationAnimaliaHymenopteraBraconidae

Fischer, 1966

9CC3E88D-0338-5416-85EC-A68B2DE968F2

Dinostigma Fischer, 1966: 182; Shenefelt 1974: 991; Wharton 1980: 38; van Achterberg 1988: 19; Yu et al. 2016.

###### Type species.

**Figure 10.**
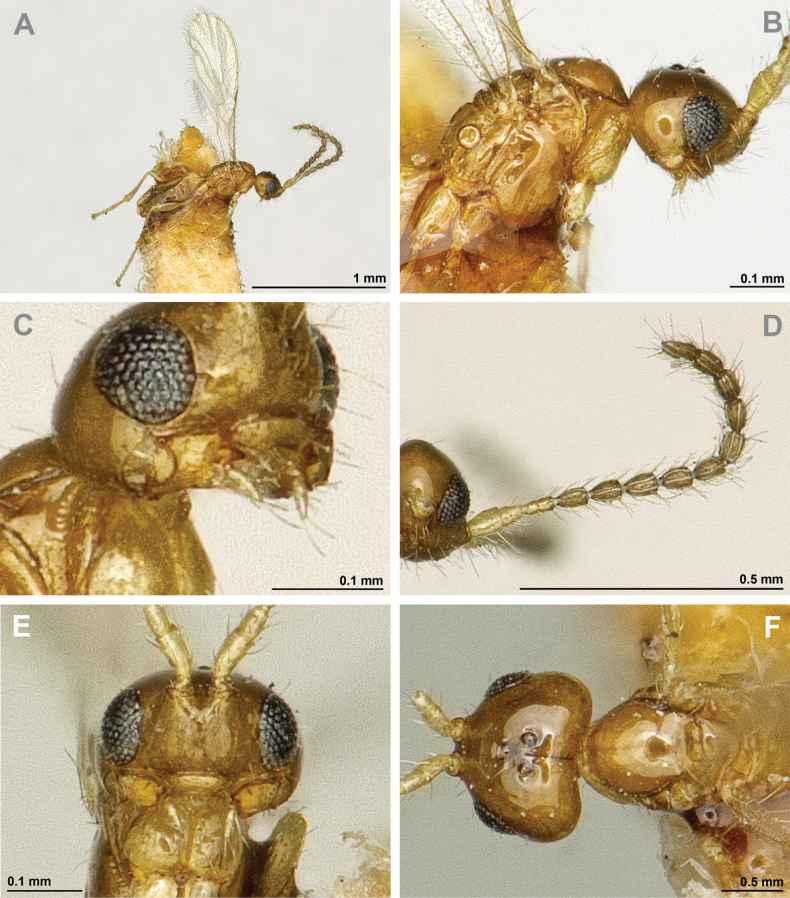
*Dinostigmamuesebecki* Fischer, 1966 (holotype, female) **A** habitus, lateral view **B** head and mesosoma, lateral view **C** mandible **D** antenna **E** head, front view **F** head and mesonotum, dorsal view.

**Figure 11.**
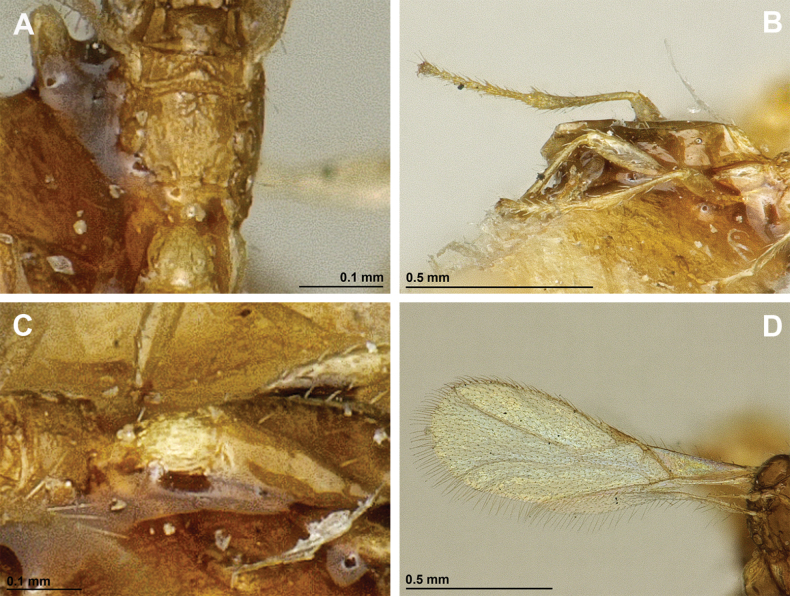
*Dinostigmamuesebecki* Fischer, 1966 (holotype, male) **A** propodeum **B** legs, metasoma and ovipositor, lateral view **C** first metasomal tergite, dorsal view **D** fore and hind wings.

*Dinostigmamuesebecki* Fischer, 1966, by monotypy (Figs 10, 11).

###### Material examined.

***Holotype*** (*Dinostigmamuesebecki*). United States Of America: • ♀, North East, Pa. [= Pennsylvania], No 9019, 6.vii.1912 (F. Johnson leg.) (NMNH).

###### Diagnosis.

Mandible small, simple, tridentate. Paraclypeal fovea short, far from reaching inner margin of eyes. Mesoscutum without mesoscutal pit; notauli present only in anterior part of mesoscutum; precoxal sulcus absent; propodeum always smooth; spiracles of propodeum large. In fore wing, marginal cell never shortened; vein r originating from basal quarter of pterostigma; vein 2-SR absent; vein cu-a postfurcal; first subdiscal cell open distally (without vein 2-1A). Hind wing with all cells open. Metasoma of ♀ more or less distinctly compressed laterally. Ovipositor sheath shorter than metasoma.

###### Remarks.

After careful revision of former *Dinostigma* and *Eudinostigma* (as subgenus of *Dinostigma*) species, only the type species of this genus, *Dinostigmamuesebecki* Fischer, 1966, is retained in *Dinostigma*. The species *D.stenosoma* (van Achterberg, 1988) is transferred to the genus *Dinotrema* as a type species of the new subgenus, Pseudoprosapha subgen. nov. (see below), because this species has the first subdiscal cell of fore wing closed, the pterostigma broad and wider than vein r length, and all cells of the hind wing closed (Dinotrema (P.) stenosoma (van Achterberg, 1988), comb. nov.).

This genus is very close to the Oriental-Afrotropical *Lysodinotrema* Fischer, 1995, because both of them share, among others, such main diagnostic cha­racters as simple tridentate mandible, short paraclypeal fovea, and mesoscutum without medio-posterior pit. However, the lack of closed cells in the hind wing in *Dinostigma* (present in *Lysodinotrema*), absence of vein 2-SR (present in *Lysodinotrema*), and absence of the precoxal sulcus (present in *Lysodinotrema*) are sufficient to separate both as different genera.

##### ﻿Genus Dinotrema

Taxon classificationAnimaliaHymenopteraBraconidae

Foerster, 1863

1B387890-40C6-56C5-B1C4-CCD4266EFE0C

Figs 12 , 13 , 14 , 15 , 16 , 17 , 18 , 19 , 20 , 21

Dinotrema Foerster, 1863: 268; Wharton 1980: 84; van Achterberg and Bin 1981: 104; van Achterberg 1988: 19; Chen and Wu 1994: 69; Wharton 2002: 56; Tobias 2003a: 138; 2004: 468; 2006: 324; Peris-Felipo et al. 2014a: 10; Yu et al. 2016; Peris-Felipo and Belokobylskij 2018a: 4.
Coloboma Foerster, 1863: 268.
Spanomeris Foerster, 1863: 268.
Synaldis Foerster, 1863: 273; Fischer 1962: 1; 1971: 139; Tobias 1971: 199 (key); Shenefelt 1974: 1020; Tobias 1986: 123; Fischer 1993b: 567; Fischer 1997: 107; Belokobylskij 2002: 404; 2004a: 1991; 2004b: 935; Belokobylskij and Tobias 2007: 58; Fischer et al. 2008: 1461; Yu et al. 2016; Peris-Felipo and Belokobylskij 2017: 4.
Scotioneurus Provancher, 1886: 156.
Alitha Cameron, 1906: 28; Shenefelt 1974: 938; van Achterberg 1988: 9; Yu et al. 2016, stat. nov.
Pterusa Fischer, 1958: 14; Shenefelt 1974: 1108; van Achterberg 1988: 50; Belokobylskij 1998a: 170; van Achterberg and Vikberg 2014: 3 (as synonym of Dinotrema Foerster); Yu et al. 2016 (as valid genus).
Aspilota auct. p.p. Fischer 1972: 327; Shenefelt 1974: 966; Fischer 1976: 345.
Carinthilota Fischer, 1975: 311; Tobias 1986: 123; van Achterberg 1988: 9; Belokobylskij 1998a: 221; Fischer 2002: 102; Yu et al. 2016, syn. nov.
Eudinostigma Tobias, 1986: 244; van Achterberg 1988: 36; Fischer 1991: 12; Fischer et al. 2006: 831; Yu et al. 2016, syn. nov.

###### Type species.

*Dinotremaerythropa* Foerster, 1863, by monotypy.

###### Diagnosis.

Mandible small, simple, tridentate, often with upper (first) tooth diminished with respect to lower (third) tooth. Paraclypeal fovea short, not reaching more than half distance between clypeus and inner margin of eyes. Mesoscutum with or without mesoscutal pit; notauli usually present only in anterior part of mesoscutum, although in some species of the subgenus Alitha it is rather well developed and reaching or almost reaching mesoscutal pit; precoxal sulcus usually present, propodeum with different types of sculpture and sometimes with longitudinal and/or transverse carinae, rarely entirely smooth. In fore wing, marginal cell never shortened; vein r originating from basal quarter of pterostigma; vein 2-SR usually present and distinctly sclerotised or sometimes (subgenus Synaldis) absent or weakly developed and vein r not angled with vein 3-SR (van Achterberg 1988); veins m-cu and cu-a postfurcal; first subdiscal cell always closed postero-apically by CU1a vein. Venation of hind wing more or less reduced, sometimes without closed cells (Zhu et al. 2017). Metasoma of ♀ more or less distinctly compressed laterally. Ovipositor sheath usually not longer than metasoma.

###### Remarks.

*Dinotrema* is the most complicated and largest genus within the tribe Alysiini with more than 440 known species, predominantly occurring in the temperate climatic regions (Peris-Felipo and Belokobyslkij 2018a). However, after studying a large amount of type material from different regions it should be possible to present a new generic classification, including the following subgenera: *Alitha* Cameron, 1906, stat. nov. (with *Carinthilota* Fischer as a new synonym), *Dinotrema* sensu stricto, *Prosapha* Foerster, 1863, Pseudoprosapha Peris-Felipo subgen. nov. and Synaldis Foerster, 1863 (with *Eudinostigma* Tobias as a new synonym).

A revision of *Eudinostigma* Tobias species was carried out for this reclassification. After careful study of the type species of *Eudinostigma* we consider it a synonym of *Dinotrema*. However, depending on the presence or absence of vein 2-SR of the fore wing, its species are divided between the subgenera *Dinotrema* and *Synaldis*. The main diagnostic characters of *Eudinostigma* are as follows: distinctly depressed head (resulting in antennal sockets situated at the upper level of eye and maximum width of head in dorsal view 1.6–2.4× width of mesoscutum), compressed mesosoma, and vein 2-SR of fore wing often absent (Tobias 1986; van Achterberg 1988). These characters also occur sometimes in *Dinotrema* species, e.g., among others, in *Dinotremabrevissimicorne* (Stelfox et Graham, 1948), *D.compressum* (Haliday, 1838), *D.parapunctatum* (Fischer, 1976), and *D.robertoi* Peris-Felipo, 2013.

The following species previously belonging to Eudinostigma are transfered to the subgenus Dinotrema sensu stricto: D. (D.) alox (van Achterberg, 1988), comb. nov.; D. (D.) entabeniense (Fischer, 2009), comb. nov.; D. (D.) latum (Chen & Wu, 1994), comb. nov.; D. (D.) planiceps (Fischer, Tormos & Pardo, 2006), comb. nov. and D. (D.) subpulvinatum (Fischer, 2009), comb. nov.. Moreover, four other Eudinostigma species are transferred to the subgenus Synaldis: D. (S.) bienesae (Fischer, Tormos & Pardo, 2006), comb. nov., D. (S.) fischeri (Tobias, 1986), comb. nov. (type species of *Eudinostigma*), D. (S.) latistigma (Fischer, 1962), comb. nov., and D. (S.) planiceps (Fischer, Tormos & Pardo, 2006), comb. nov.

Furthermore, after studying the types of *Dinostigma* and *Eudinostigma* species, we consider the features of *Eudinostigmastenosoma* van Achterberg, 1988 (see below) enough different to transfer it to a new subgenus Pseudoprosapha subgen. nov.: Dinotrema (Pseudoprosapha) stenosoma (van Achterberg, 1988), comb. nov.

In summary, five subgenera of the genus *Dinotrema* are recognised, *Alitha* Cameron, 1906, stat. nov., *Dinotrema* sensu stricto, *Prosapha* Foerster, 1863, Pseudoprosapha Peris-Felipo subgen. nov. and Synaldis Foerster, 1863.

##### ﻿ Subgenus Alitha

Taxon classificationAnimaliaHymenopteraBraconidae

Cameron, 1906, stat. nov.

67364353-1597-50F9-9EC5-DAC33FF9535F

Alitha Cameron, 1906: 28; van Achterberg 1988: 9.
Carinthilota Fischer, 1975, syn. nov.

###### Type species.

*Alithalongipennis* Cameron, 1906, by monotypy (lost).

###### Material examined.

**Figure 12.**
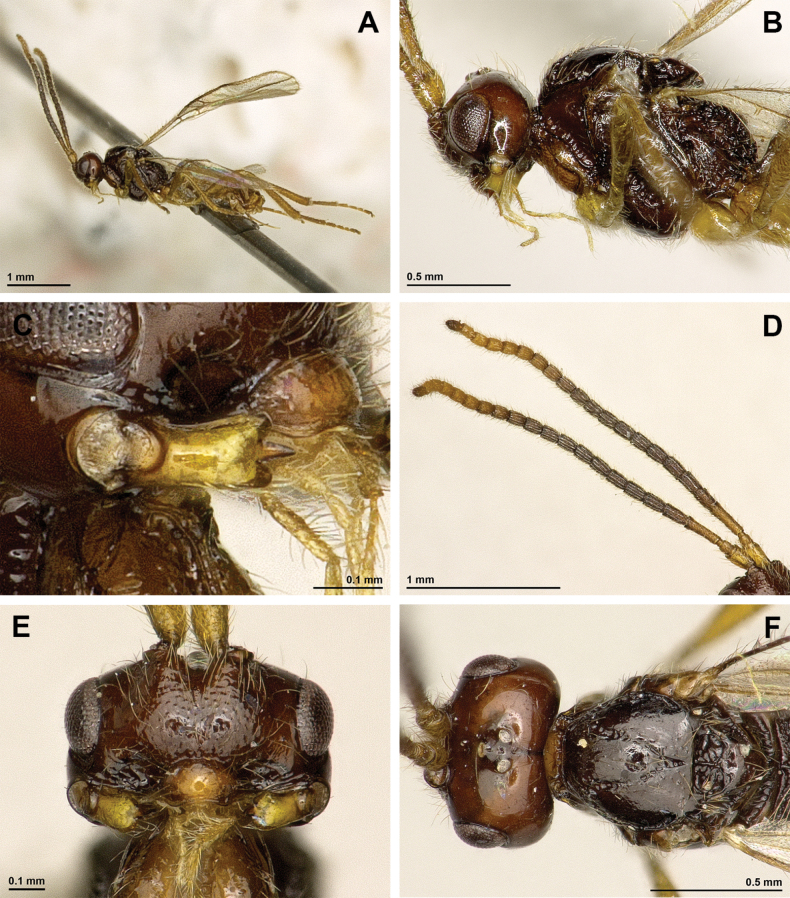
Dinotrema (Alitha) parapsidalis (Fischer, 1975), comb. nov. (holotype, female) **A** habitus, lateral view **B** head and mesosoma, lateral view **C** mandible **D** antenna **E** head, front view **F** head and mesonotum, dorsal view.

**Figure 13.**
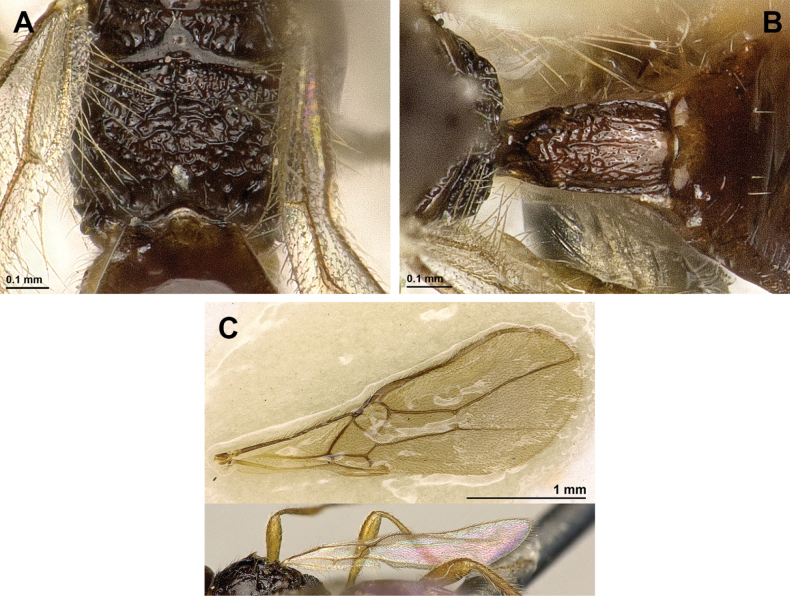
Dinotrema (Alitha) parapsidalis (Fischer, 1975), comb. nov. (holotype, female) **A** propodeum **B** first metasomal tergite, dorsal view **C** fore and hind wings.

***Holotype*** (*Carinthilotaparapsidalis*) (Figs 12, 13) Austria: • ♀, Kärnten (88), 1 km O. Heft b(ei) Hüttenberg, 1000–1100 m, 25.viii.1973 (Fischer leg.) (NHMW). ***Holotype*** (*Carinthilotavechti*) The Netherlands: • ♀, Putten (Gld.), Malaise trap, 24–28.ix.1970 (J.v.d. Vecht leg.) (RMNH).

###### Diagnosis.

This subgenus has all main characters of *Dinotrema* sensu stricto but differs from it by having the notauli more or less complete posteriorly, reaching or almost reaching the mesoscutal pit.

###### Remarks.

Despite the loss of the type material of *Alitha* Cameron, 1906 described from South Africa (van Achterberg 1988) and thanks to the relative complete description of this genus given by Cameron (1906) and the additional comments by van Achterberg (1988), Alitha is considered a subgenus of Dinotrema (stat. nov.). Moreover, the genus *Carinthilota* Fischer, 1975 is considered a junior synonym of the subgenus Alitha (syn. nov.) because both share identical diagnostic characters. Unfortunately, so far only four Palaearctic and Oriental species are known and no studied specimens from the Afrotropical region.

The development of the notauli in *Alitha* species is highly variable: usually they are developed as rows of closely circular grooves more or less reaching the medio-posterior mesoscutal pit [Dinotrema (A.) longipennis (Cameron, 1906), comb. nov., D. (A.) parapsidalis (Fischer, 1975), comb. nov. and D. (A.) vechti (van Achterberg, 1988), comb. nov.] while the distal part of the notauli is more or less reduced in the two Eastern Palaearctic species [D. (A.) lada (Belokobylskij, 1998), comb. nov. and D. (A.) mavka (Belokobylskij, 1998), comb. nov.]. The variable development of the notauli supports our opinion that the presence of nearly complete notauli in several genera of *Aspilota* group (*Dinotrema* and *Orthostigma*) is an unsuitable generic character; at most it may be used provisionally at subgeneric level.

##### ﻿ Subgenus Dinotrema

Taxon classificationAnimaliaHymenopteraBraconidae

Foerster, 1863, sensu stricto

5A301192-5696-5C54-A5C0-9EAED80B64D9

Dinotrema Foerster, 1863: 268; Wharton 1980: 84; van Achterberg and Bin 1981: 104; van Achterberg 1988: 19; Chen and Wu 1994: 69; Wharton 2002: 56; Tobias 2003a: 138; 2004: 468; 2006: 324: Peris-Felipo et al. 2014a: 10; Yu et al. 2016; Peris-Felipo and Belokobylskij 2018a: 4.
Pterusa Fischer, 1958: 14; van Achterberg and Vikberg 2014: 1, 3.

###### Type species.

**Figure 14.**
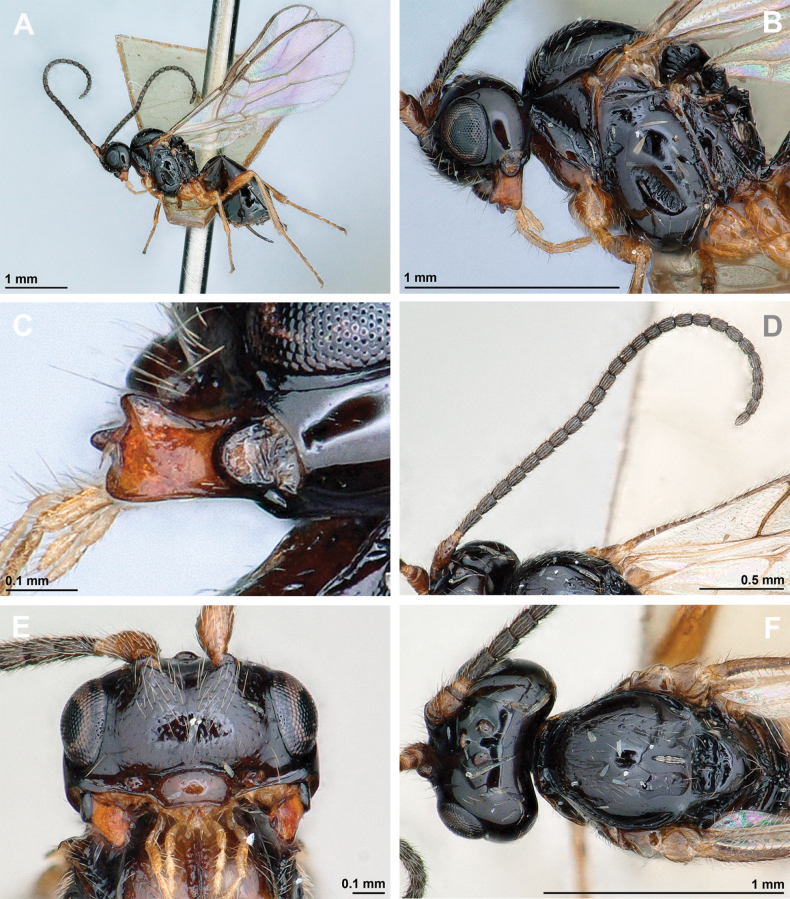
Dinotrema (Dinotrema) erythropum Foerster, 1863 (female) **A** habitus, lateral view **B** head and mesosoma, lateral view **C** mandible **D** antennae **E** head, front view **F** head and mesonotum, dorsal view.

**Figure 15.**
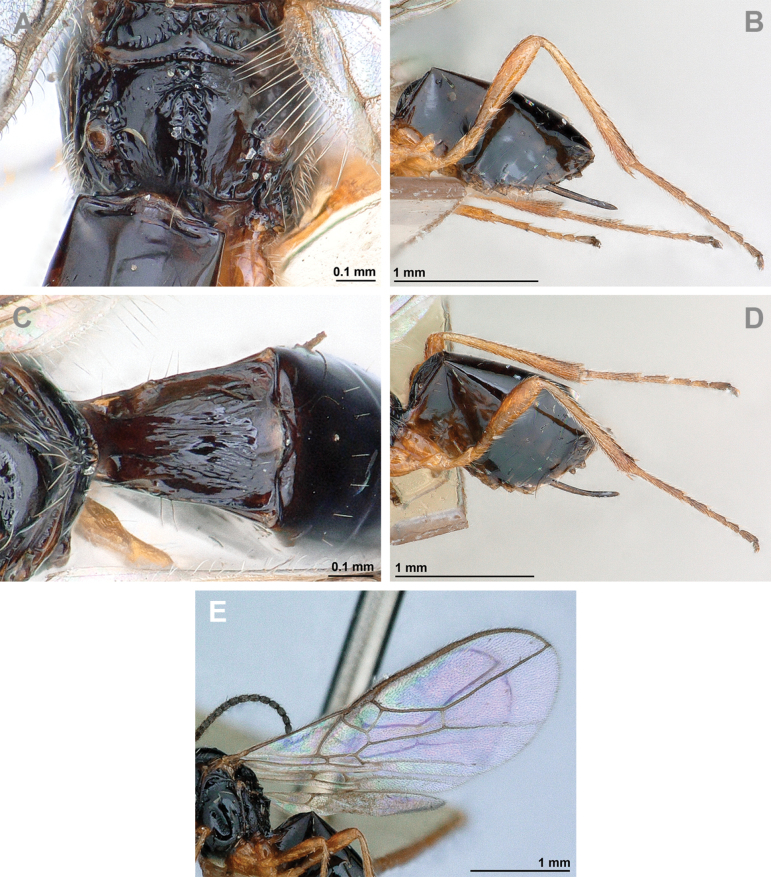
Dinotrema (Dinotrema) erythropum Foerster, 1863 (female) **A** propodeum, dorsal view **B** hind leg, lateral view **C** first metasomal tergite, dorsal view **D** metasoma and ovipositor, lateral view **E** fore and hind wings.

*Dinotremaerythropum* Foerster, 1863, by original designation (Figs 14, 15).

###### Material examined

(Dinotrema (Dinotrema) erythropum): England: • 1 female (paratype of *Aspilotapraecipua*) and 1 male (paratype id.), Coll. Marshall Cornwall, Botusfleming (HNHM). Denmark: • 1 female, E-Jylland, Frisenborg, 28.vii.1986 (Munk leg.) (NMA). Finland: • 1 female, Sa. Valkeala, 6772:483, 28.vii.1977 (Koponen leg.) (NMA); • 1 female, same locality but, 29.vii.1977 (NMA). Hungary: • 1 female, Ugod, Somberek Hubertlak-Kórnyéle, 26–29.vi.1967 (Papp leg.) (HNHM). Luxembourg: • 2 females, Tratten, b. Murau Stmk. Coll Fulmek, 14.viii.1942 and 13.x.1954 (NHMW). Netherlands: • 1 female, Wijster (Dr.) opposite Biological Station, 22–30.ix.1975 (van Achterberg leg.) (RMNH). Spain: • 1 female, Valencia, 16.vii.1942 (NHMW).

###### Diagnosis.

The main diagnostic characters of this subgenus are the short paraclypeal fovea which remain far from the eye margins, the pterostigma very narrow (linear) and vein 2-SR of fore wing present and more or less completely sclerotised.

###### Remarks.

This is the largest subgenus including the main part of *Dinotrema* species with approx. 440 species described worldwide (Peris-Felipo and Belokobyslkij 2018a). As shown by van Achterberg and Vikberg (2014), *Pterusa* Fischer, 1958 is a synonym of *Dinotrema* sensu stricto, because any differences are absent in females, and it is based only on the brachyptery of the males.

##### ﻿ Subgenus Prosapha

Taxon classificationAnimaliaHymenopteraBraconidae

Foerster, 1863

E8DBE58A-8E99-5AC3-999D-6723F4043C7E

Figs 16 , 17

Prosapha Foerster, 1863: 263; Shenefelt 1974: 1018; Tobias 1986: 121.
Dinotrema (Prosapha) van Achterberg 1988: 88 (as synonym of Dinotrema); Tobias 2003b: 810; Belokobylskij and Tobias 2007: 11; van Achterberg and Vikberg 2014: 3; Yu et al. 2016.

###### Type species.

**Figure 16.**
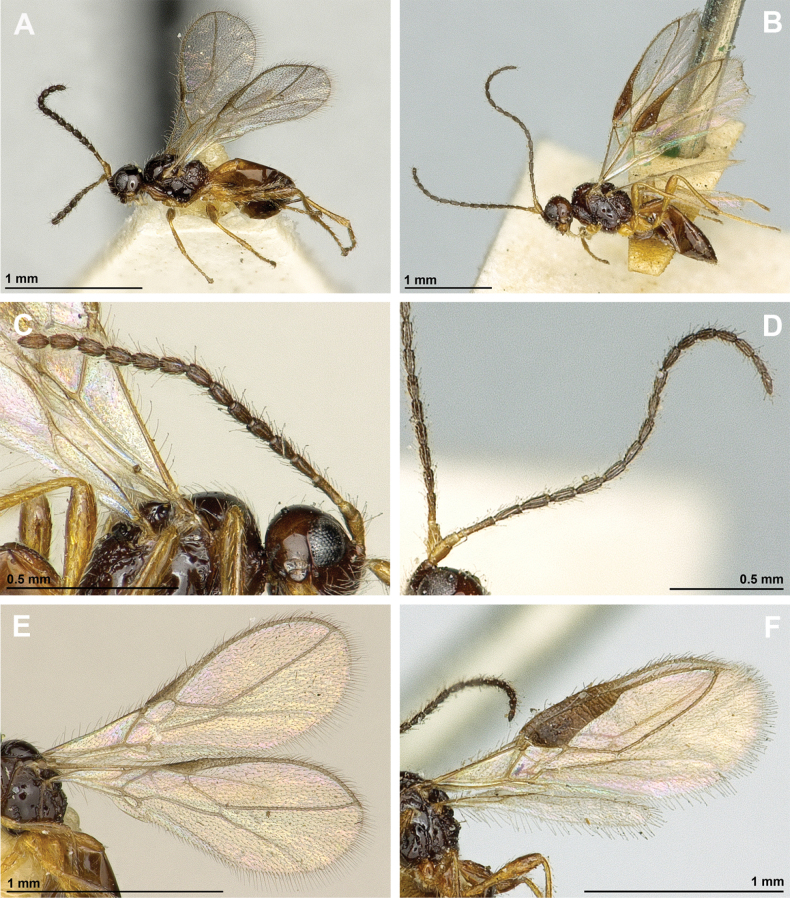
Dinotrema (Prosapha) speculum (Haliday, 1838) (**A, C, E**: female; **B, D, F**: male) **A, B** habitus, lateral view **C, D** antenna **E, F** fore and hind wings.

**Figure 17.**
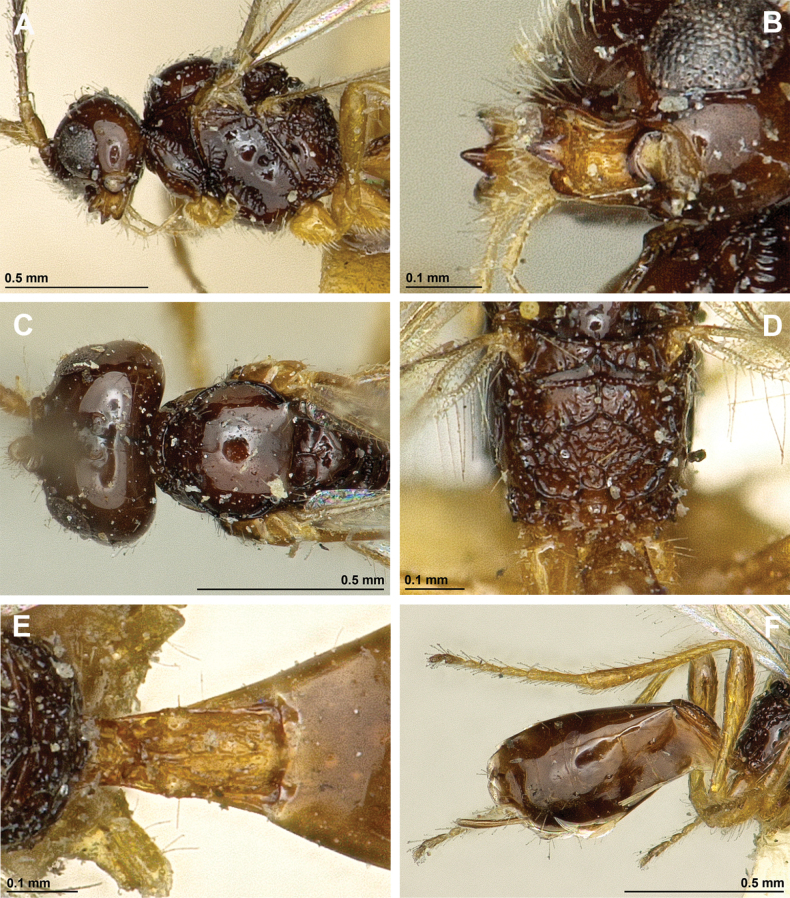
Dinotrema (Prosapha) speculum (Haliday, 1838) (female) **A** head and mesosoma, lateral view **B** mandible **C** head and mesonotum, dorsal view **D** propodeum **E** first metasomal tergite, dorsal view **F** legs, metasoma and ovipositor, lateral view.

*Alysiaspeculum* Haliday, 1838, by original designation (Figs 16, 17).

###### Material examined

(Dinotrema (Prosapha) speculum): Austria: • ♂, Spitzzicken, Burgenland, 24.viii.1959 (Fischer leg.) (NHMW). Russia: • 8 ♂, Leningradskaya Province, Tolmachevo, 23.viii.1960 (Tobias leg.) (ZISP); • 2 ♂, Novgorod Province, 20 km NW of Pestovo, 6.vii.1986 (Tobias leg.) (ZISP); • ♀, ibid, 15.viii.1990 (ZISP); • ♀, ibid, 27.vii.1999 (ZISP); • ♀, ibid, 1.viii.1999 (ZISP); • ♀, ibid, 5.viii.2001 (ZISP); • ♀, Volgograd Province, 10 km S of Novokhopersk, 10.vii.1977 (Tobias leg.) (ZISP); • ♂, Krasnodar Territory, Sochi, Lazarevskoe, 30.v.1988 (Tobias leg.) (ZISP); • ♂, Chelyabinsk Province, Ilmenskiy Nature Reserve, 17.vii.1958 (Tobias leg.) (ZISP).

###### Diagnosis.

This subgenus shares the main characters of *Dinotrema* sensu stricto but differs by having, in the fore wing, the maximum width of pterostigma wider than vein r (especially in males) and vein 2-SR of fore wing always present.

###### Remarks.

This subgenus includes five Palaearctic species: D. (P.) comptum Tobias, 2003, D. (P.) pachysemoides Tobias, 2003, D. (P.) speculum (Haliday, 1838), D. (P.) tobiasi (Fischer, 1994) and D. (P.) ussuricum Tobias, 2007. The status of *Prosapha* has been variable for a long time. Foerster (1863) described this genus based on the distinctive large, cuneiform and heavily sclerotised pterostigma of the male. Van Achterberg (1988) and Tobias (2003b) considered *Prosapha* species inside of *Dinotrema* based on their morphological similarity and because *Prosapha* females possess a narrower pterostigma weakly se­parated from the metacarp (1-R1). *Prosapha* species can be differentiated from Pseudoprosapha subgen. nov. by the presence of vein 2-SR (which is absent in *Pseudoprosapha*).

##### ﻿ Pseudoprosapha Peris-Felipo, subgen. nov.

Taxon classificationAnimaliaHymenopteraBraconidae

957FCD6C-7C23-54DC-8089-A8C55CE46C70

https://zoobank.org/5526942A-61D5-4085-B080-BCB05D629900

###### Type species.

**Figure 18.**
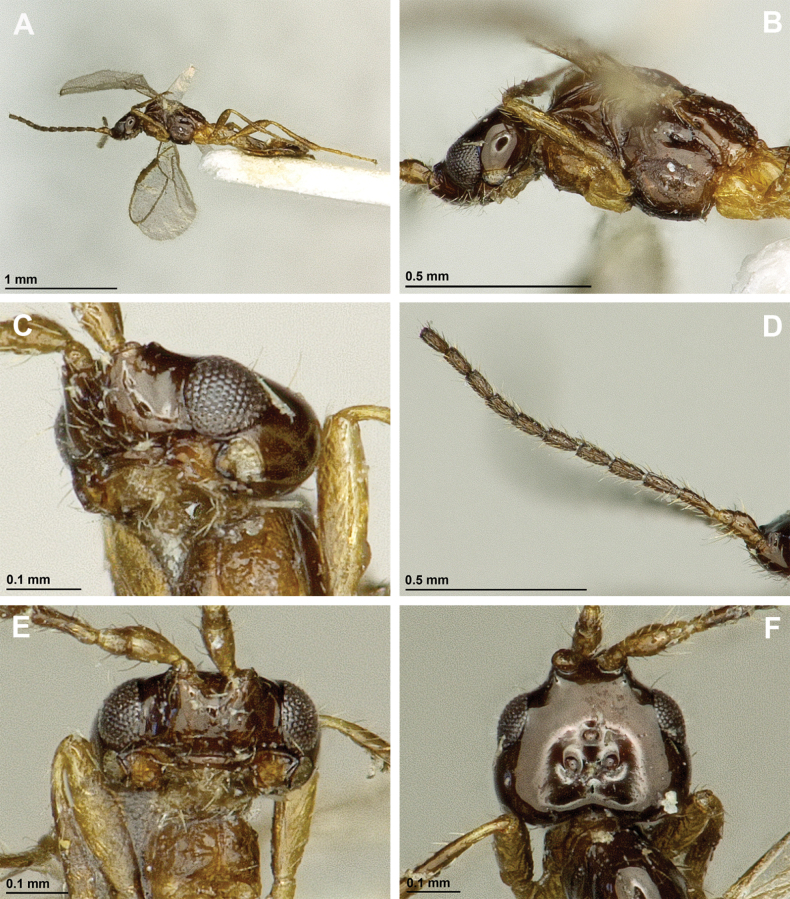
Dinotrema (Pseudoprosapha) stenosoma (van Achterberg, 1988), comb. nov. (holotype, male) **A** habitus, lateral view **B** head and mesosoma, lateral view **C** mandible **D** antenna **E** head, front view **F** head, dorsal view.

**Figure 19.**
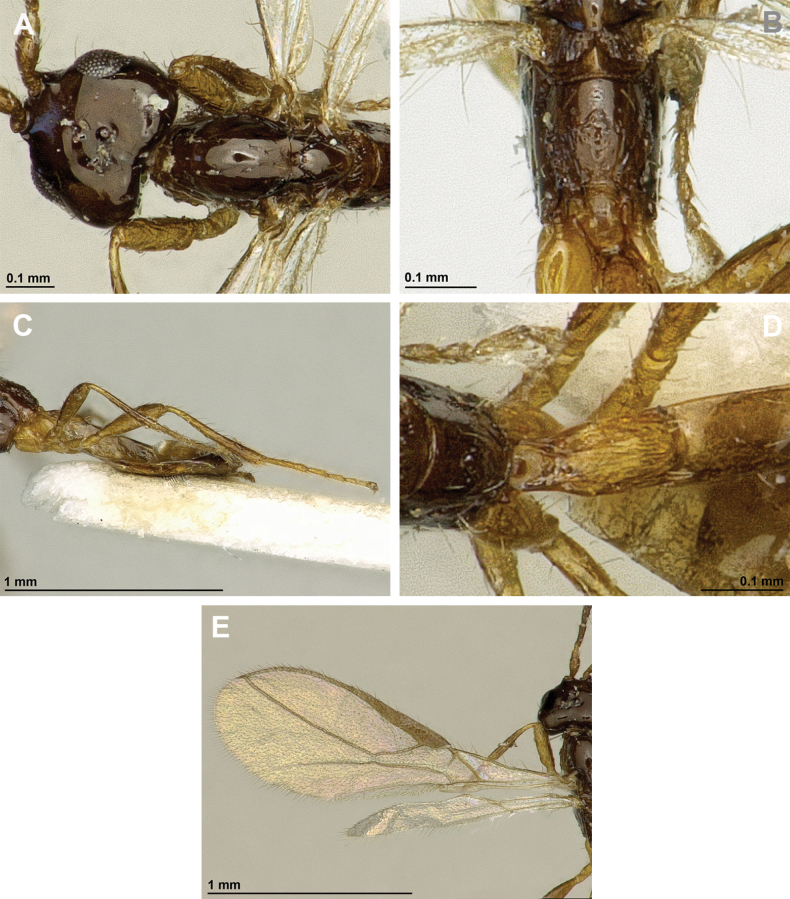
Dinotrema (Pseudoprosapha) stenosoma (van Achterberg, 1988), comb. nov. (holotype, male) **A** head and mesonotum, dorsal view **B** propodeum **C** legs and metasoma, lateral view **D** first metasomal tergite, dorsal view **E** fore and hind wings.

*Eudinostigmastenosoma* van Achterberg, 1988 (Figs 18, 19).

###### Material examined.

***Holotype*** (*Eudinostigmastenosoma*) Hungary: • ♂, Budapest, Biró, 21.ix.1927, “226” (RMNH).

###### Diagnosis.

Mandible small, simple, tridentate, with upper (first) tooth diminished with respect to lower (third) tooth. Paraclypeal fovea short, length not more than half distance between clypeus and inner margin of eyes. Mesoscutum without medio-posterior pit; notauli present only in anterior part of mesoscutum; precoxal sulcus present; propodeum completely smooth. Marginal cell of fore wing never shortened; vein r originating from basal quarter of wide pterostigma; vein 2-SR absent; vein cu-a postfurcal; first subdiscal cell always closed postero-apically by vein CU1a. Hind wing with all cells closed. Metasoma of ♀ more or less distinctly compressed posteriorly. Ovipositor sheath not longer than metasoma.

###### Remarks.

This new subgenus includes only a single species, Dinotrema (Pseudoprosapha) stenosoma (van Achterberg, 1988), comb. nov.. This subgenus shares with *Prosapha* the comparatively broad pterostigma (viz., wider than the length of vein r) and in female vein r + 3-SR forming a (nearly) straight line but differs by the loss of vein 2-SR (present in *Prosapha*), the depressed head (antennal sockets situated near the upper level of the eyes), the strongly compressed mesosoma and the very narrow clypeus. These differences make it worth to name a different subgenus for it.

##### ﻿ Subgenus Synaldis

Taxon classificationAnimaliaHymenopteraBraconidae

Foerster, 1863

97A112E2-02A3-5F63-AA22-76BC2E11900C

Synaldis Foerster, 1863: 273; Fischer 1962: 1; 1971: 139; Tobias 1971: 199 (key); Shenefelt 1974: 1020; Tobias 1986: 123; Fischer 1993b: 567; 1997: 107; Belokobylskij 2002: 404; 2004a: 1991; 2004b: 935; Belokobylskij and Tobias 2007: 58; Fischer et al. 2008: 1461; Yu et al. 2016; Peris-Felipo and Belokobylskij 2017: 4; Zhu et al. 2017: 61; Dias de Oliveira and Penteado-Dias 2024: 280.
Eudinostigma Tobias, 1986: 244, syn. nov.

###### Type species.

**Figure 20.**
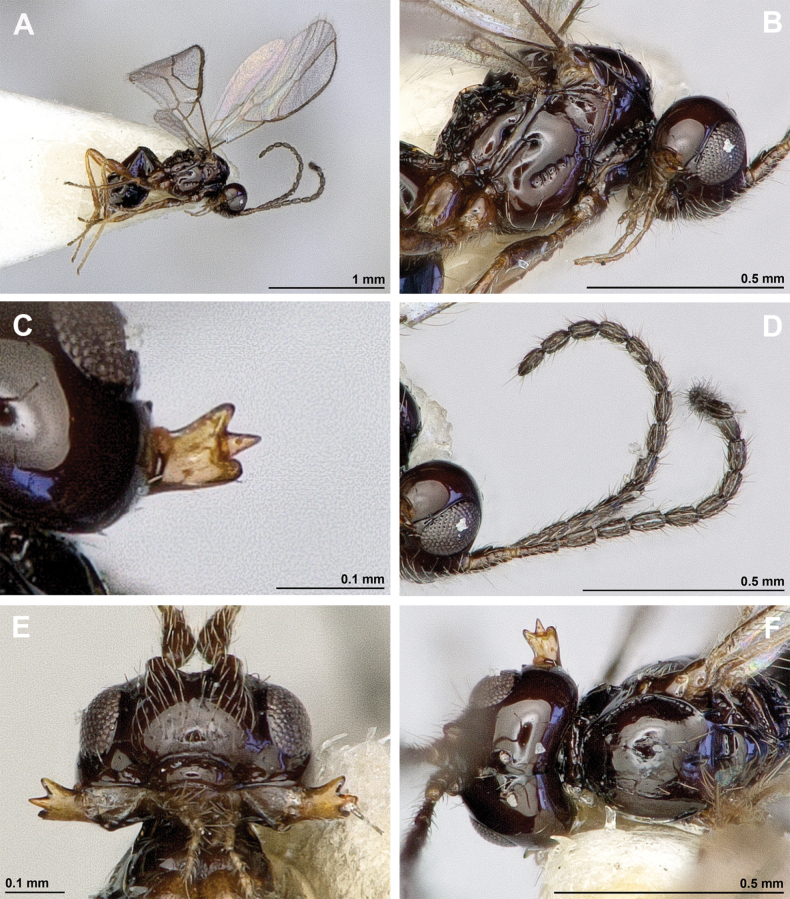
Dinotrema (Synaldis) concolor (Nees, 1812) (female) **A** habitus, lateral view **B** head and mesosoma, lateral view **C** mandible **D** antenna **E** head, front view **F** head and mesonotum, dorsal view.

**Figure 21.**
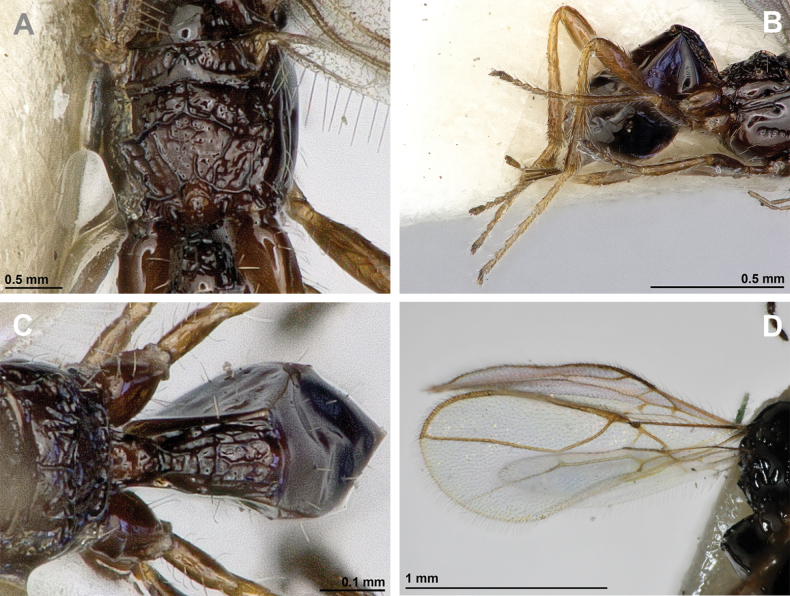
Dinotrema (Synaldis) concolor (Nees, 1812) (female) **A** propodeum **B** legs, metasoma and ovipositor, lateral view **C** first metasomal tergite, dorsal view **D** fore and hind wings.

*Bassusconcolor* Nees, 1812, by original designation (lost) (Figs 20, 21).

###### Material examined.

Dinotrema (Synaldis) concolor: Austria: • ♀, Mischerdorf, Burgenland, 5.ix.1956 (Fischer leg.) (NHMW); • ♀, Mischerdorf, Burgenland, 6.viii.1958 (Fischer leg.) (NHMW); • ♀, Spitzzicken, Burgenland, 5.ix.1956 (Fischer leg.) (NHMW). Hungary: • ♀, Nagyrákos, 6.vi.1985 (Rozner leg.) (HNHM). Slovakia: • ♀, Orosva Polhora, 25.vii.1988 (Podlussány leg.) (HNHM). Dinotrema (Synaldis) cracipes [= *Pterusacracipes*]: ***Holotype***: Austria: • ♂, Wimpassing, Nieder-Österreich (Leitha-Gebirge), 2.v.1915 (Fischer leg.) (NHMW).

###### Diagnosis.

The main characters of this subgenus are shared with *Dinotrema* sensu stricto, but it has vein 2-SR of fore wing absent.

###### Remarks.

The status of *Synaldis* has been uncertain for a long time. Van Achterberg (1988) revised the genera of the *Aspilota* group and first sy­nonymised this genus with the re-established *Dinotrema* based on the not enlarged paraclypeal fovea (the plesiomorphic state). As a result, the former *Synaldis* species were distributed among the genera *Aspilota* and *Dinotrema* according to the new used diagnostic feature, the size and position of the paraclypeal fovea. For some time, the synonymy of *Synaldis* was not accep­ted by several experts working on alysiine taxa (Fischer 1993a, 1993b; Papp 2001; Belokobylskij 2002; Peris-Felipo et al. 2014a). It is necessary to underline that the apomorphic feature of the subgenus, the complete reduction of vein 2-SR of the fore wing, is a peculiar evolutionary event which also resulted in the disappearance of the distinct break (corner) between veins r and 3-SR and this part is only gently and relatively widely curved. Such an apomorphic state of the wing venation might represent an important qualitative transformation and could support at least a subgeneric status of *Synaldis* (Belokobylskij 2002; Peris-Felipo and Belokobylskij 2014, 2017). However, the intermediate state is also known, both with the presence of non-sclerotised vein 2-SR and vein r not angled with vein 3-SR (e.g., D. (D.) pulvinatum (Stelfox & Graham) as depicted by van Achterberg 1988) or vein 2-SR entirely absent (e.g., D. (S.) cespitator (Belokobylskij, 2004), comb. nov.), D. (S.) perfidum (Fischer, 1970), comb. nov. (as depicted by Fischer 1970) and D. (S.) trematosum (Fischer, 1967), comb. nov. (as depicted by Fischer 1967) with vein r weakly angled with 3-SR). The variation of vein 2-SR from entirely absent to entirely present and non-sclerotised vein is aptly shown in D. (D.) concinnum (Haliday, 1838). Therefore, we agree with Zhu et al. (2017) to recognise *Synaldis* as a subgenus for convenience. Its position as separate genus likely will render the genus *Dinotrema* paraphyletic, and the loss of vein 2-SR occurred probably more than once in *Dinotrema* and is variable within some taxa as illustrated by *D.concinnum* (König 1972) and the type species of the genus *Synaldotrema* (Belokobylskij and Tobias 2002).

##### ﻿Genus Leptotrema

Taxon classificationAnimaliaHymenopteraBraconidae

van Achterberg, 1988

B795D419-168B-50CB-8D97-5474F4202BBC

Leptotrema van Achterberg, 1988: 42; Chen and Wu 1994: 94; Belokobylskij 1998a: 219; Fischer 2002: 102; Yu et al. 2016.

###### Type species.

**Figure 22.**
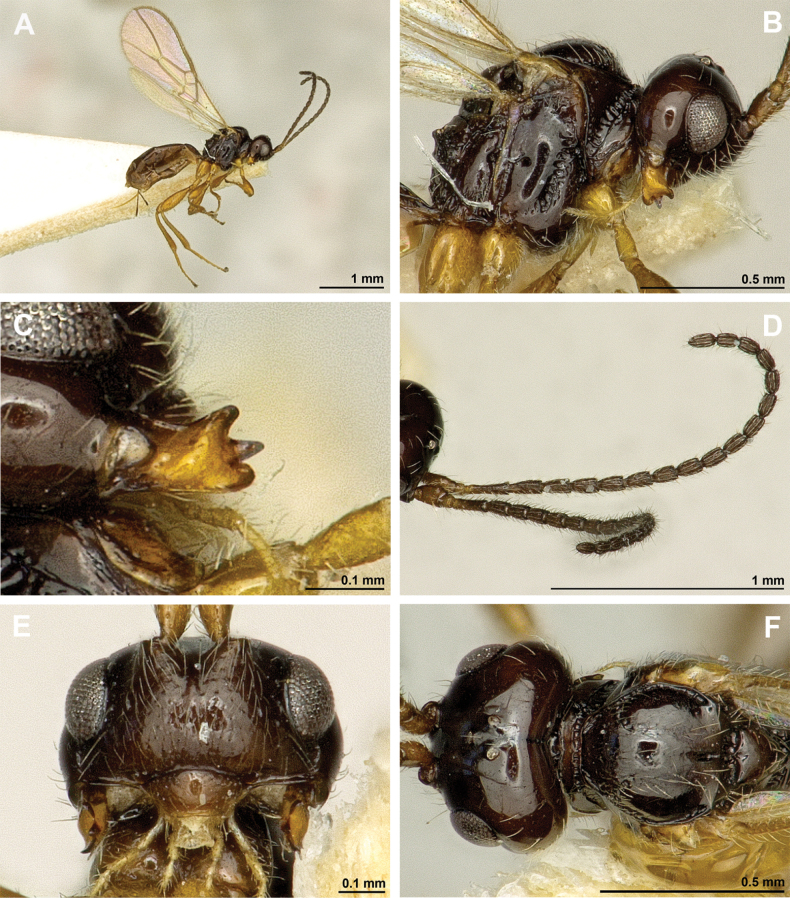
*Leptotremadentifemur* (Stelfox, 1943) (female) **A** habitus, lateral view **B** head and mesosoma, lateral view **C** mandible **D** antenna **E** head, front view **F** head and mesonotum, dorsal view.

**Figure 23.**
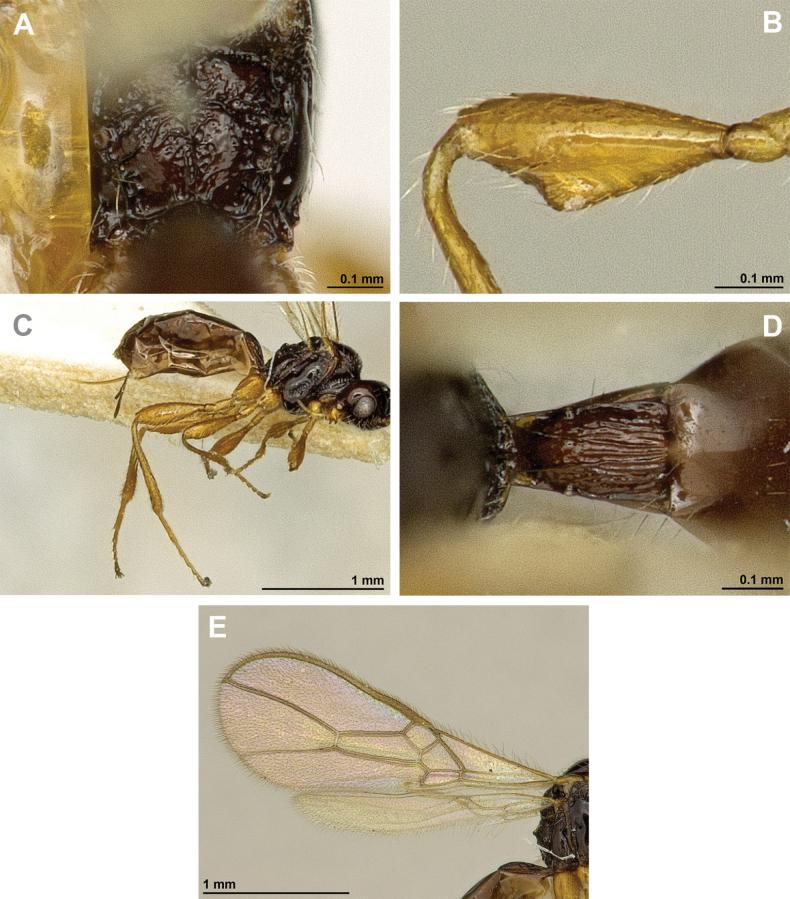
*Leptotremadentifemur* (Stelfox, 1943) (female) **A** propodeum **B** fore femur, lateral view **C** legs, metasoma and ovipositor, lateral view **D** first metasomal tergite, dorsal view **E** fore and hind wings.

*Aspilotadentifemur* Stelfox, 1943, by original designation (Figs 22, 23).

###### Material examined.

(*Leptotremadentifemur*) Denmark: • ♀, Stegelykke VG, 8–15.vii.1991 (Munk leg.) (PFEC). The Netherlands: • ♀, Putten (Gld.), Malaise trap, 26.ix–2.x.1973 (J.v.d. Vecht leg.) (RMNH).

###### Diagnosis.

Mandible small, simple, tridentate, often with upper (first) tooth diminished with respect to lower (third) tooth. Paraclypeal fovea short, not reaching more than half distance between clypeus and inner margin of eyes. Mesoscutum with or without mesoscutal pit; notauli usually present only in anterior part of mesoscutum; precoxal sulcus usually present, propodeum with different types of sculpture and sometimes with longitudinal and/or transverse carinae, rarely entirely smooth. Fore femur has a distinct apomorphic character, viz., the presence of a large obtuse tooth (flange) or two or three small teeth. In fore wing, marginal cell never shortened; vein r originating from basal quarter of pterostigma; vein 2-SR usually present and distinctly sclerotised; veins m-cu and cu-a postfurcal; first subdiscal cell always closed postero-apically by vein CU1a. Metasoma of ♀ more or less distinctly compressed laterally. Ovipositor sheath usually not longer than metasoma.

###### Remarks.

Only three rare *Leptotrema* species are known from the Palaearctic, Oriental and Australasian regions, *L.bovefemora* (Bhat, 1979), *L.dentifemur* (Stelfox, 1943) and *L.wilhelmense* Braet & van Achterberg, 2014. Wharton (2002) treated Leptotrema only as subgenus of Dinotrema, but some other experts (Fischer 2002; Belokobylskij and Tobias 2007; Braet and van Achterberg 2014) preferred to consider it as a valid genus based on its unique apomorphic character: the presence of the ventral tooth or teeth of the fore tibia. The study based on the main morphological characters show that *Leptotrema* deserves generic status.

##### ﻿Genus Lysodinotrema

Taxon classificationAnimaliaHymenopteraBraconidae

Fischer, 1995

A9E2A851-87A7-5BA8-A228-84DB734E080C

Lysodinotrema Fischer, 1995: 717; Fischer 2002: 103; Yu et al. 2016.

###### Type species.

**Figure 24.**
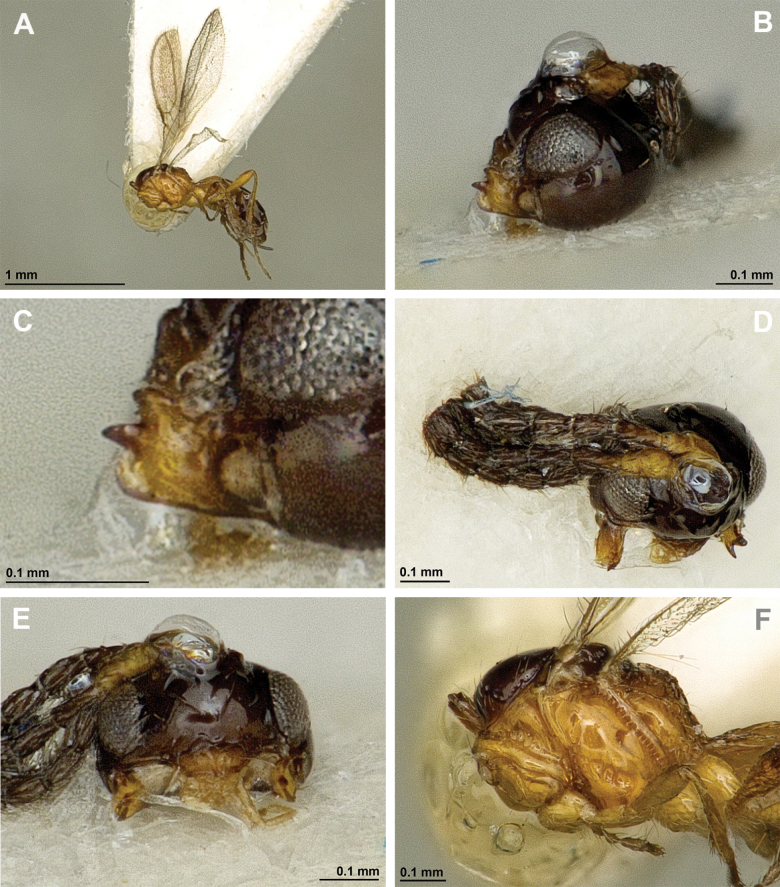
*Lysodinotremamadli* Fischer, 1995 (holotype, female) **A** habitus, lateral view **B** head, lateral view **C** mandible **D** antenna **E** head, front view **F** mesosoma, lateral view.

**Figure 25.**
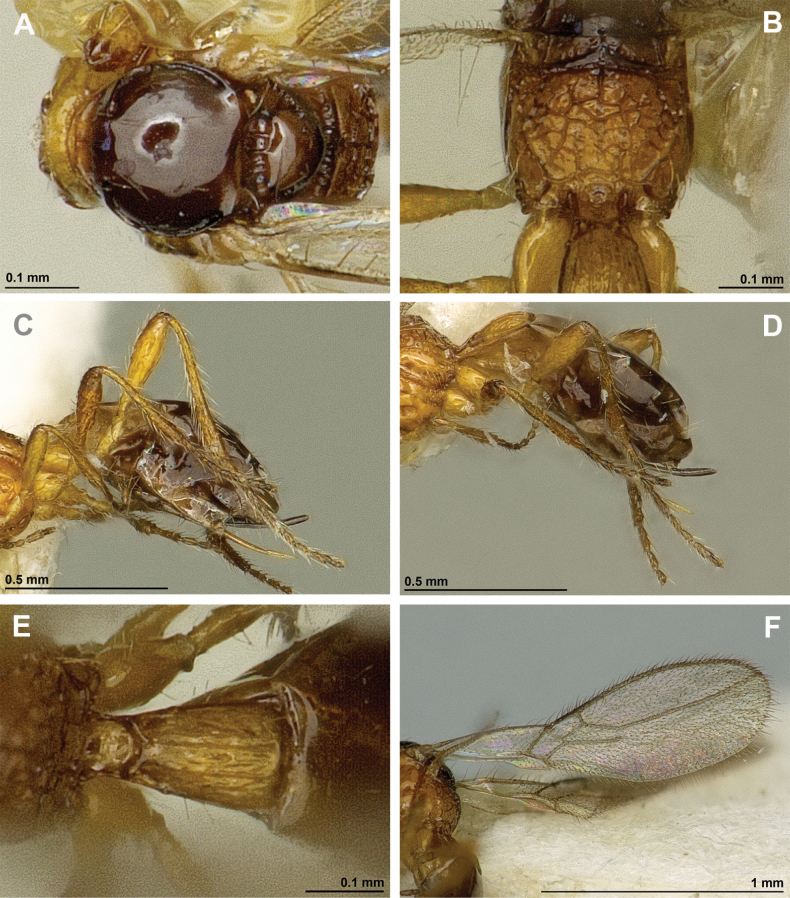
*Lysodinotremamadli* Fischer, 1995 (holotype, female) **A** mesonotum, dorsal view **B** propodeum **C** hind leg, lateral view **D** metasoma and ovipositor, lateral view **E** first metasomal tergite, dorsal view **E** fore and hind wings.

*Lysodinotremamadli* Fischer, 1995, by original designation (Figs 24, 25).

###### Material examined.

***Holotype*** (*Lysodinotremamadli*) Madagascar: • ♀, Ste. Marie Flues Manandriana, 14–25.xi.1994 (Fischer leg.) (NHMW).

###### Diagnosis.

Mandible small, simple, tridentate. Paraclypeal fovea short, remaining far from the inner margin of eyes. Mesoscutum without mesoscutal pit; notauli present only in anterior half of mesoscutum; precoxal sulcus present; propodeum mainly sculptured, without areola. In fore wing, marginal cell never shortened; vein r originating from basal quarter of pterostigma; vein 2-SR present and sclerotised; veins m-cu and cu-a always postfurcal; first subdiscal cell completely open posteriorly and without vein 2-1A. Hind wing without closed cells. Metasoma of ♀ more or less distinctly compressed. Ovipositor sheath shorter than metasoma.

###### Remarks.

This rare genus, with only three described species from the tro­pical areas (*L.madli* Fischer, 1995, *L.minimum* Fischer, 2004, *L.sarawakense* Fischer, 1995), is considered to be related with *Dinostigma* Fischer, 1966, because of sharing the open first subdiscal cell in the fore wing. However, the combination of closed cells in the hind wing (absent in *Dinostigma*), presence of vein 2-SR of fore wing (absent in *Dinostigma*) and of the precoxal sulcus (absent in *Dinostigma*) makes it possible to maintain *Lysodinotrema* as a valid genus.

##### ﻿Genus Panerema

Taxon classificationAnimaliaHymenopteraBraconidae

Foerster, 1863

84C1B202-8A33-5F01-99F6-2BC27AC79082

Panerema Foerster, 1863: 263; Szépligeti 1904: 203; van Achterberg 1988: 47; Fischer 2002: 102; Belokobylskij and Kula 2012: 43; van Achterberg and Vikberg 2014: 3; Yu et al. 2016.

###### Type species.

**Figure 26.**
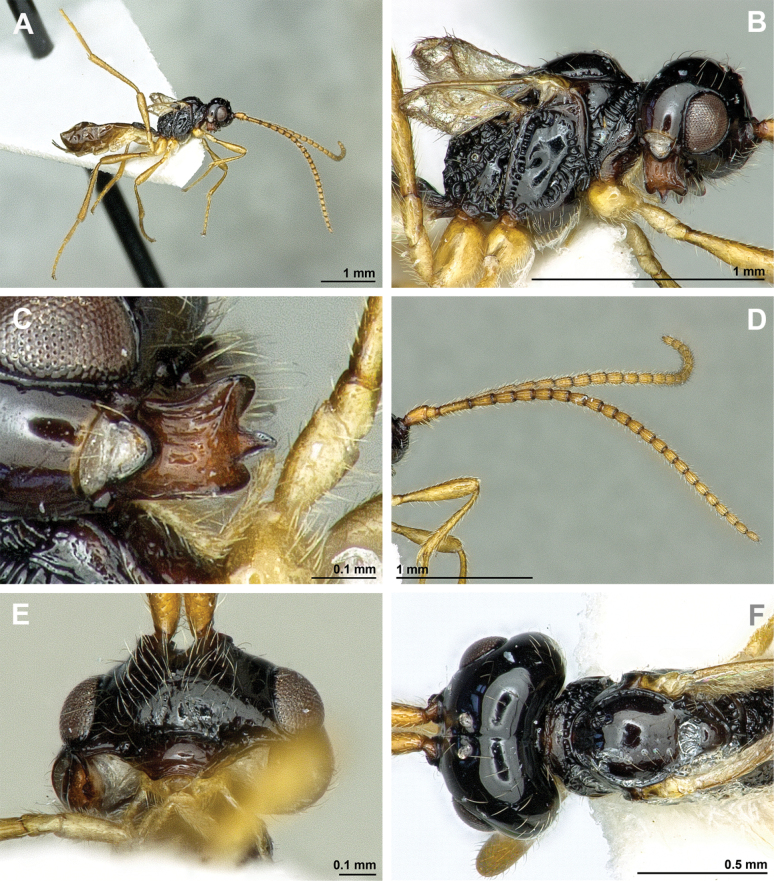
*Paneremainops* Foerster, 1863, comb. nov. (female) **A** habitus, lateral view **B** head and mesosoma, lateral view **C** mandible **D** antenna **E** head, front view **F** head and mesonotum, dorsal view.

**Figure 27.**
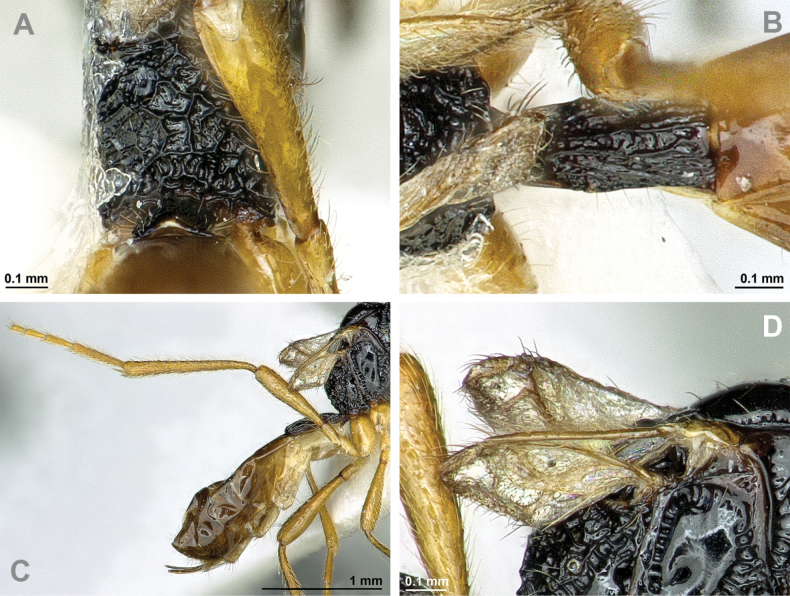
*Paneremainops* Foerster, 1863, comb. nov. (female) **A** propodeum **B** first metasomal tergite, dorsal view **C** legs, metasoma and ovipositor, lateral view **D** fore wing.

*Paneremainops* Foerster, 1863, by original designation (lost) (Figs 26, 27).

###### Material examined.

(*Paneremainops*): Germany: • ♀, Zaarensee, Seggenwiese, 29.vi.1998 (v. Broen leg.) (PFEC). The Netherlands: • ♀, Cadier, 5.v.1975 (B. v. Aartsen leg.) (RMNH).

###### Diagnosis.

Mandible small, simple, tridentate, often with upper (first) tooth diminished with respect to lower (third) tooth. Paraclypeal fovea short, not reaching more than half distance between clypeus and inner margin of eyes. Third antennal segment distinctly elongated. Mesoscutum with or without mesoscutal pit; notauli usually present only in anterior part of mesoscutum. Scutellum with a transverse crenulate depression subposteriorly. Females are brachypterous with strongly reduced wings (commonly in this group of genera males are brachypterous but females macropterous). The preserved distal anterior veins in such wing are distinctly thickened, with veins r and 2-SR of the fore wing absent but hind wing with closed cells (van Achterberg 1988; Belokobylskij and Kula 2012). Metasoma of ♀ more or less distinctly compressed laterally. Ovipositor sheath usually not longer than metasoma.

###### Remarks.

During many years, *Panerema* was considered as a valid genus (van Achterberg 1988; Fischer 2002; Belokobylskij and Tobias 2007; Belokobylskij and Kula 2012). As shown by van Achterberg (1988) despite its uncertain position of this taxon it has two synapomorphies, viz., the scutellum has a transverse crenulate depression subposteriorly and the third antennal segment is at least 1.5× longer than the fourth segment. The value of both characters is uncertain (although perhaps apomorphic), but the diagnostic character study carried out shows that *Panerema* deserves the status of genus due to its distance from other genera.

##### ﻿Genus Synaldotrema

Taxon classificationAnimaliaHymenopteraBraconidae

Belokobylskij & Tobias, 2002, stat. nov.

B71C09E8-8BBA-53F3-ADAF-EA0D1951D4B4

Synaldotrema Belokobylskij & Tobias, 2002: 3 (as subgenus of Dinotrema Foerster); Belokobylskij and Tobias 2007: 11; van Achterberg and Vikberg 2014: 3; Yu et al. 2016.

###### Type species.

**Figure 28.**
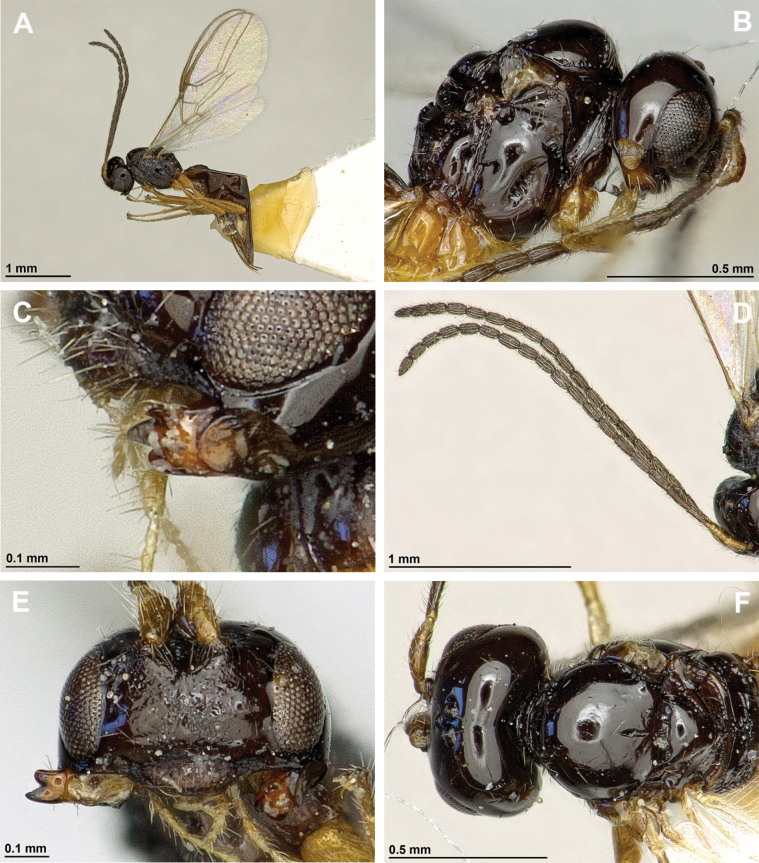
*Synaldotremaspeciosum* Belokobylskij & Tobias, 2002, comb. nov. (holotype, female) **A** habitus, lateral view **B** head and mesosoma, lateral view **C** mandible **D** antenna **E** head, front view **F** head and mesonotum, dorsal view.

**Figure 29.**
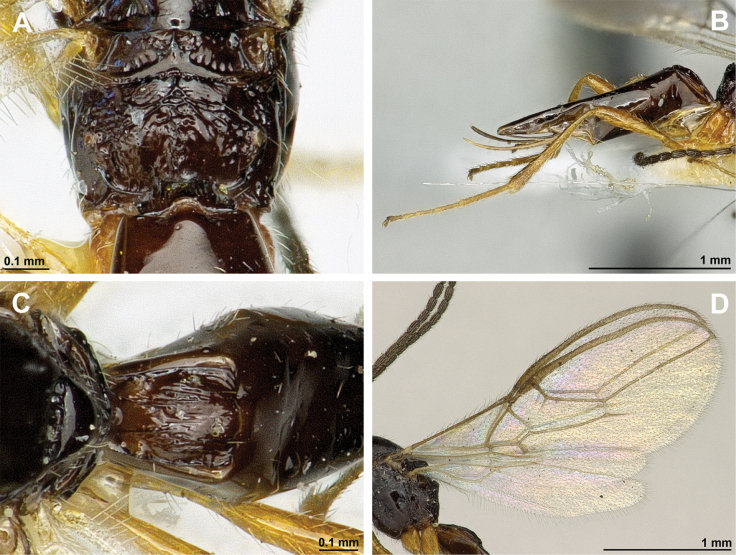
*Synaldotremaspeciosum* Belokobylskij & Tobias, 2002, comb. nov. (holotype, female) **A** propodeum **B** legs, metasoma and ovipositor, lateral view **C** first metasomal tergite, dorsal view **D** fore and hind wings.

Dinotrema (Synaldotrema) speciosum Belokobylskij & Tobias, 2002, by original designation (Figs 28, 29).

###### Material examined.

***Holotype*** (Dinotrema (Synaldotrema) speciosum) Russia: • ♀, Primorskiy kray, 10 km SSW of Partizansk, border of forest, 12–13.vii.1996 (S. Belokobylskij) (ZISP). ***Paratypes*** (Dinotrema (Synaldotrema) speciosum) Russia: • ♀, Primorskiy kray, 50 km N of Olga, mixed forest, 29.vii.1979 (S. Belokobylskij) (ZISP); • ♀, Primorskiy kray, Pogranichnyi District, Barabash-Levada, forest, 3–6.vi.1980 (S. Belokobylskij) (ZISP); • ♀, Primorskiy kray, 42 km S of Plastun, forest, 24.vi.1979 (S. Belokobylskij) (ZISP); • ♀, Republic of Tuva, 14 km E of Kyzyl, lowland of Ka Khem River, 31.v.1975 (D. Kasparyan) (ZISP).

###### Diagnosis.

*Synaldotrema* Belokobylskij & Tobias shares the main characters of *Dinotrema* sensu stricto but differs by having the hypopygium of the female strongly retracted under the posterior tergites of metasoma and the fourth tergite strongly elongated, ~ 2.5× longer than fifth tergite (vs approximately of equal length in *Dinotrema* s. str.).

###### Remarks.

The type species of *Synaldotrema* (*D.speciosum* Belokobylskij & Tobias, 2002) has a variable vein 2-SR of the fore wing. This vein is usually present, but sometimes, mostly discoloured and its posterior half or sometimes entire vein 2-SR is absent (Belokobylskij and Tobias 2002). Previous studies showed that the value of the reduction of this vein illustrates well the subgeneric division (Belokobylskij and Tobias 2002), however the current diagnostic character study proved that retraction of hypopygium has enough value to consider *Synaldotrema* as a valid genus.

#### ﻿*Orthostigma* group

##### ﻿Genus Cubitalostigma

Taxon classificationAnimaliaHymenopteraBraconidae

Fischer, 1998

AA4133A9-07C5-57BC-89E4-7CF149DFF3DF

Cubitalostigma Fischer, 1998: 482; Fischer 2002: 100; Yu et al. 2016.

###### Type species.

**Figure 30.**
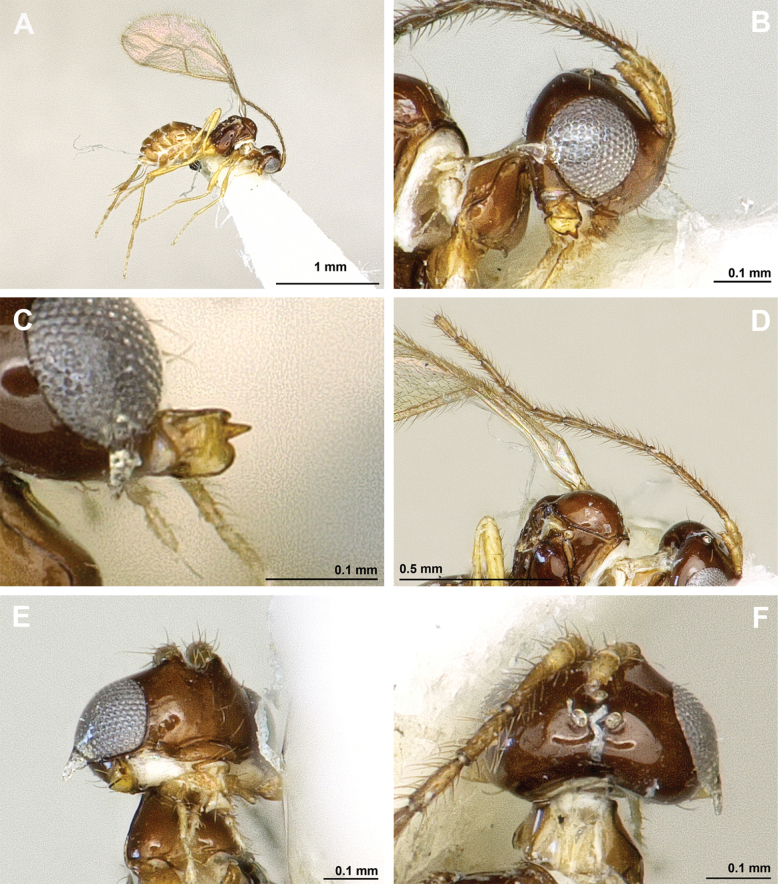
*Cubitalostigmareichli* Fischer, 1998 (holotype, female) **A** habitus, lateral view **B** head, lateral view **C** mandible **D** antenna **E** head, front view **F** head, dorsal view.

**Figure 31.**
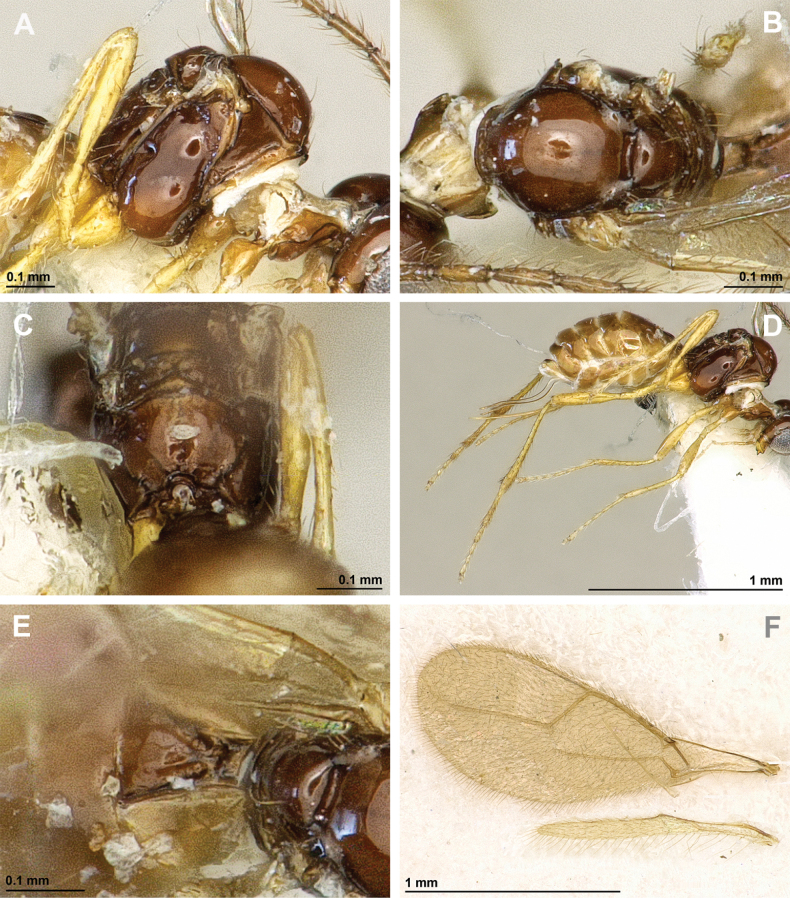
*Cubitalostigmareichli* Fischer, 1998 (holotype, female) **A** mesosoma, lateral view **B** mesonotum, dorsal view **C** propodeum **D** legs, metasoma and ovipositor, lateral view **E** first metasomal tergite, dorsal view **D** fore and hind wings.

*Cubitalostigmareichli* Fischer, 1998, by monotypy (Figs 30, 31).

###### Material examined.

***Holotype*** (*Cubitalostigmareichli*) Indonesia: • ♀, Sumatra, Aceh, Gunung Leuser Nat. Pk., Ketambe Res. Sta., 1° rainforest, Mature forest, Terrace 4 closed canopy, 400 m, 3°41'N, 97°29'E, Malaise trap W/pans, 1–30.xi.1989 (D.C. Darling leg.) (NHMW).

###### Diagnosis.

Mandible small, simple, tridentate, with upper (first) tooth diminished with respect to lower (third) tooth, with complete submedial transverse curved carina. Paraclypeal fovea short, remaining far removed from edge of eyes. Mesoscutum without mesoscutal pit; notauli present only in anterior part of mesoscutum; precoxal sulcus always absent; propodeum smooth. In fore wing, marginal cell never shortened; vein r originating from almost middle of pterostigma; first submarginal cell very reduced; second submarginal cell widened; vein 2-SR present and well sclerotised; first subdiscal cell closed postero-apically by CU1a vein. Subbasal cell of hind wing minute but closed. Metasoma of ♀ distinctly compressed laterally. Ovipositor sheath shorter than metasoma.

###### Remarks.

This is a peculiar monotypic genus with only the type species known from Indonesia. *Cubitalostigma* is characterised by the very aberrant venation of the fore wing, with vein r arising almost from the middle of the very narrow pterostigma, very far from its basal part. This is a unique character within the subtribe Aspilotina.

##### ﻿Genus Neorthostigma

Taxon classificationAnimaliaHymenopteraBraconidae

Belokobylskij, 1998

E32AB647-052F-51E8-8809-BA67BE2237D2

Neorthostigma Belokobylskij, 1998b: 9; Fischer 2001: 65; Wharton 2002: 91 (as subgenus); Belokobylskij and Tobias 2007: 10 (as valid genus); Yu et al. 2016; Belokobylskij et al. 2019: 215; Peris-Felipo et al. 2020: 33; Dias de Oliveira and Penteado-Dias 2023: 481.

###### Type species.

**Figure 32.**
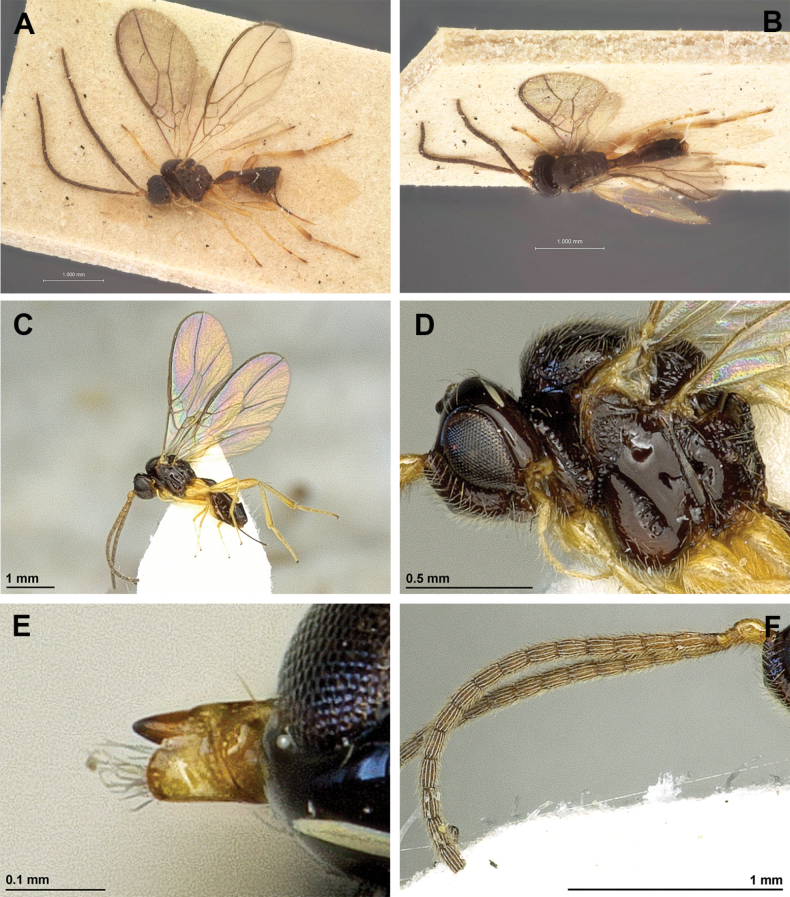
*Neorthostigmamacrops* (Stelfox & Graham, 1951) (**A, B**: female, holotype of *A.macrops*; **C–F**: female, holotype of *Neorthostigmaeoum*) **A, C** habitus, lateral view **B** habitus, dorsal view **D** head and mesosoma, lateral view **E** mandible **F** antenna.

**Figure 33.**
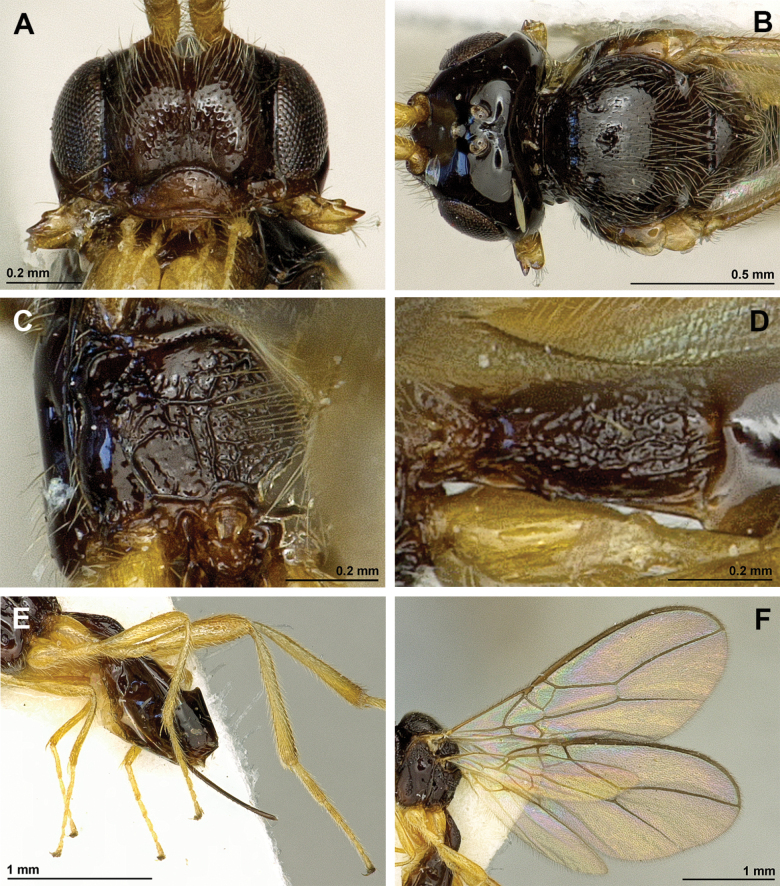
*Neorthostigmamacrops* (Stelfox & Graham, 1951) (female, holotype of *Neorthostigmaeoum*) **A** head, front view **B** head and mesonotum, dorsal view **C** propodeum, dorsal view **D** first metasomal tergite, dorsal view **E** hind leg, metasoma and ovipositor, lateral view **F** fore and hind wings.

*Neorthostigmaeoum* Belokobylskij, 1998 (= *Aspilotamacrops* Stelfox & Graham, 1951), by original designation (Figs 32, 33) [synonymised by Peris-Felipo et al. 2020].

###### Material examined.

***Holotype*** (*Aspilotamacrops*) Ireland: • ♀, Sligo, S. shore of Lough Gill near Doonee Rock, 15.x.1937 (AWS leg.) [USNM #76022; USNMENT01569377] (NMNH). ***Holotype*** (*Neorthostigmaeoum* (= *macrops*)) Russia: • ♀, Primorskiy kray, Anisimovka, forest, glades, 16.viii.1979 (S. Belokobylskij leg.) (ZISP). ***Paratypes*** (*Neorthostigmaeoum* (= *macrops*)) Russia: • 2 ♀, Primorskiy kray, Spassk-Dal’niy, forest, glades, 16 and 22–23.viii.1995 (S. Belokobylskij leg.) (ZISP); • 1 ♀, Sakhalin Island, 10 km W of Aniva, mixed forest, 15.viii.1981 (S. Belokobylskij leg.) (ZISP). Japan: • 1 ♂, Fukuoka, Nogochi, Fukuoka-shi, 28.viii.1992 (V. Makarkin leg.) (ZISP).

###### Additional studied material.

Norway: • 1 ♀, Oslo [AK], Maridalen, Dausjøen, Spruce forest, 5.vi–16.x.2010, 60.01234 N 10.787665 E, 160 m, Malaise trap, river outlet (Lars Ove Hansen leg.) (NHMO). Russia. Leningradskaya Province: • 1 ♀, Tolmachevo, mixed forest, 22.viii.1960 (V. Tobias leg), “*Aspilotamacrops* Stelf., Tobias det. 1961” (ZISP). Primorskiy kray: • 1 ♀, 30 km E of Spassk-Dal’niy, forest, glades, 4.vi.1984 (S. Belokobylskij leg.) (ZISP); • 1 ♀, Nadezhdinskiy District, 15 km SSW of Nezhino, forest, 16–18.vii.1993 (S. Belokobylskij leg.) (ZISP); • 1 ♀, 30 km SE of Ussuriysk, forest, border of forest, 12–17.vii.2001 (S. Belokobylskij leg.) (ZISP); • 1 ♀, Vladivostok, Okeanskaya, forest, 25.vii.2001 (S. Belokobylskij leg.) (ZISP); • 1 ♀, Vladivostok, Sedanka, forest, border of forest, 30.vii.2001 (S. Belokobylskij leg.) (ZISP).

###### Diagnosis.

Mandible small, tridentate, with very small and screwed upper tooth, with complete transverse and curved submedian carina. Paraclypeal fovea long, reaching or almost reaching inner margin of eyes. Mesoscutum always without mesoscutal pit; notauli present only in anterior half of mesoscutum; precoxal sulcus always developed; propodeum with wide and rather distinctly delineated by carina areola and with different types of sculpture but sometimes almost smooth. In fore wing, marginal cell never shortened; vein r originating from basal quarter of pterostigma; vein 2-SR always developed; veins m-cu and cu-a postfurcal; first subdiscal cell always closed postero-apically by vein CU1a. Metasoma of ♀ more or less distinctly compressed. Ovipo­sitor sheath shorter than metasoma.

###### Remarks.

Five described species are known: one from the Palaearctic region (widely distributed from Ireland to Japan), two from Papua New Guinea (Peris-Felipo et al. 2020) and two from Brazil (Dias de Oliveira and Penteado-Dias 2023). This genus is closely related to *Orthostigma* according to its specialised mandibles. Wharton (2002) treated *Neorthostigma* as a subgenus of *Orthostigma*. However, the combination of such important diagnostic characters such as the absence of eye-antennal socket sulcus, the large paraclypeal fovea reaching or almost reaching inner margin of eye, the usual absence of mesoscutal pit, the face and sometimes the mesoscutum entirely covered by dense setosity distinctly indicate a separate position of this taxon at the genus level (Belokobylskij et al. 2019; Peris-Felipo et al. 2020). Its hosts are still unknown.

##### ﻿Genus Orthostigma

Taxon classificationAnimaliaHymenopteraBraconidae

Ratzeburg, 1844

8F55F37A-359B-5410-9A2B-F02015796AC4

Figs 34 , 35 , 36 , 37 , 38 , 39 , 40 , 41

Orthostigma Ratzeburg, 1844: 53; Königsmann 1969: 2; Shenefelt 1974: 997; Wharton 1980: 85; Tobias 1986: 117; van Achterberg 1988: 44; Chen and Wu 1994: 99; Fischer 1995: 670; Belokobylskij 1998a: 209; Fischer 2002: 102; Wharton 2002: 91; Belokobylskij and Tobias 2007: 10; Yu et al. 2016; Zhu et al. 2017: 68.
Africostigma Fischer, 1995: 677 (as subgenus of Orthostigma); Yu et al. 2016; Peris-Felipo and Belokobylskij 2020: 411.
Patrisaspilota Fischer, 1995: 721; Fischer 2002: 102; 2004: 78; 2010: 636; Wharton 2002: 91 (as subgenus of Orthostigma); Yu et al. 2016; Peris-Felipo et al. 2019: 366; Peris-Felipo and Belokobylskij 2020: 412.
Whartonstigma Peris-Felipo in Peris-Felipo and Belokobylskij 2020: 412 (as subgenus of Orthostigma).

###### Type species.

*Aphidiusflavipes* Ratzeburg, 1844: 71, by monotypy.

###### Synonyms.

*Delocarpa* Foerster, 1863; *Ischnocarpa* Foerster, 1863; *Patrisaspilota* Fischer, 1995; *Africostigma* Fischer, 1995; *Whartonstigma* Peris-Felipo, 2020.

###### Diagnosis.

Mandible small, tridentate and with a wide ventral lobe as third tooth, with complete submedial transverse curved carina. Paraclypeal fovea short, far distant from inner margin of eyes. First flagellar segment usually longer or sometimes as long as second flagellar segment (slightly shorter in subgenus Africostigma). Mesoscutum usually with mesoscutal pit; notauli often present only in anterior part of mesoscutum, but in subgenus Patrisaspilota notauli almost reaching mesoscutal pit; precoxal sulcus always present; propodeum usually with different types of sculpture and sometimes with longitudinal or transverse carinae, rarely almost smooth. In fore wing, marginal cell never shortened; vein r originating from basal quarter of pterostigma; vein 2-SR usually distinctly sclerotised (but absent in subgenus Whartonstigma); veins m-cu and cu-a postfurcal; first subdiscal cell always closed postero-apically by vein CU1a. Metasoma of ♀ more or less distinctly laterally compressed. Ovipositor sheath usually not longer than metasoma.

###### Remarks.

This genus includes more than 60 described species and is easily separated from other genera in the *Aspilota* group by the presence of the peculiar structure of mandible with complete transverse and curved submedial carina and usually wide lobe-shaped third tooth.

Currently four subgenera are recognised within this genus, *Africostigma* Fischer, 1995, *Orthostigma* sensu stricto, *Patrisaspilota* Fischer, 1995, and *Whartonstigma* Peris-Felipo, 2020 (Peris-Felipo and Belokobylskij 2020).

##### ﻿ Subgenus Africostigma

Taxon classificationAnimaliaHymenopteraBraconidae

Fischer, 1995

F6FE184F-6AC4-5EF1-8FC2-EBD6694D8F1D

Africostigma Fischer, 1995: 677 (as subgenus of Orthostigma); Yu et al. 2016; Peris-Felipo and Belokobylskij 2020: 411.

###### Type species.

**Figure 34.**
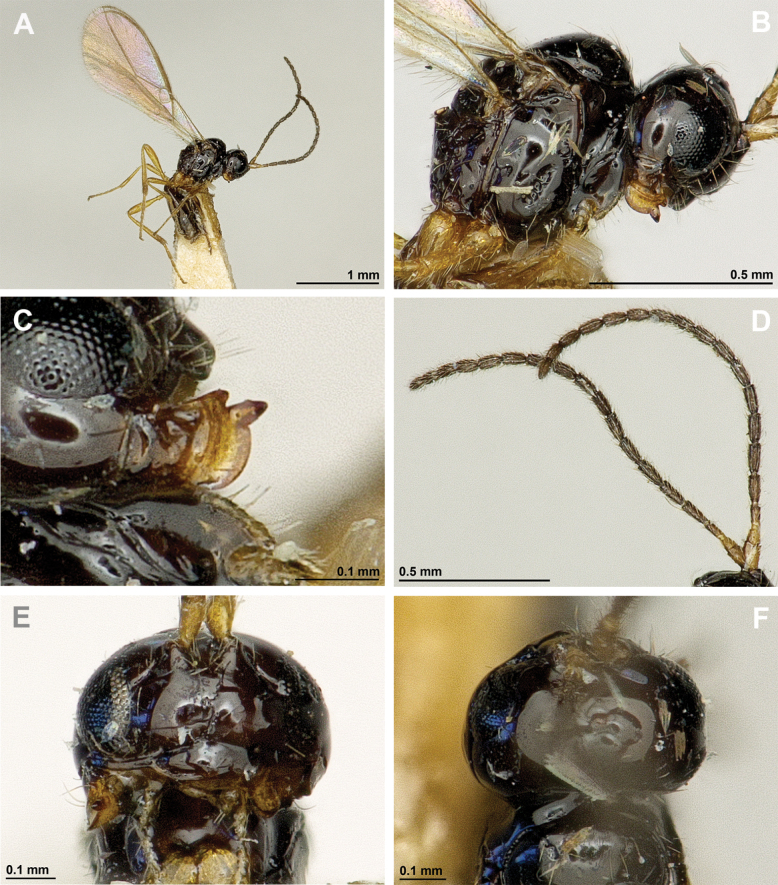
Orthostigma (Africostigma) karkloofense Fischer, 1995 (holotype, female) **A** habitus, lateral view **B** head and mesosoma, lateral view **C** mandible **D** antenna **E** head, front view **F** head, dorsal view.

**Figure 35.**
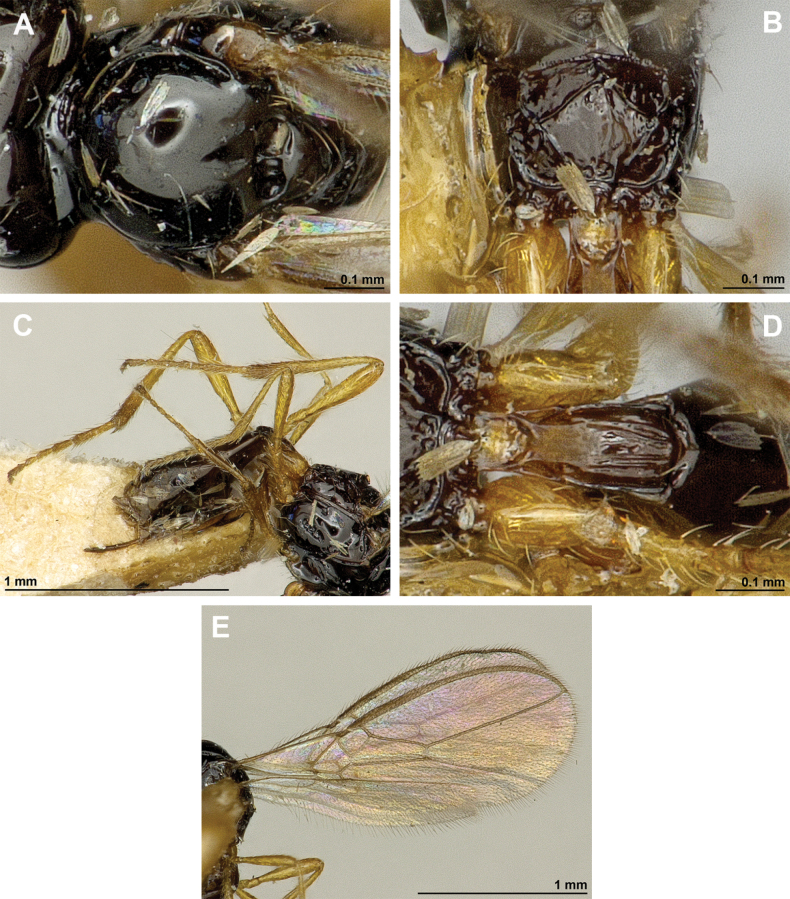
Orthostigma (Africostigma) karkloofense Fischer, 1995 (holotype, female) **A** mesonotum, dorsal view **B** propodeum **C** legs, metasoma and ovipositor, lateral view **D** first metasomal tergite, dorsal view **E** fore and hind wings.

Orthostigma (Africostigma) karkloofense Fischer, 1995, by original designation (Figs 34, 35).

###### Material examined.

***Holotype*** (Orthostigma (Africostigma) karkloofense) South Africa: • ♀, Howick, Natal, Karkloof Forest, 19.ix.1963 (Haeselbarth leg.) (ZSSM).

###### Diagnosis.

This Afrotropical subgenus includes two species from South Africa and shares the main diagnostic characters with *Orthostigma* but differs from all other subgenera by having the first flagellar segment of antenna shorter than the second one.

##### ﻿ Subgenus Orthostigma

Taxon classificationAnimaliaHymenopteraBraconidae

sensu stricto

EAEC1556-ACE8-5134-8130-D9A0B573E38C

Figs 36 , 37

Orthostigma Ratzeburg, 1844: 53; Königsmann 1969: 2; Shenefelt 1974: 997; Wharton 1980: 85; Tobias 1986: 117; van Achterberg 1988: 44; Chen and Wu 1994: 99; Fischer 1995: 670; Belokobylskij 1998a: 209; Fischer 2002: 102; Wharton 2002: 91; Belokobylskij and Tobias 2007: 10; Yu et al. 2016; Zhu et al. 2017: 68; Peris-Felipo and Belokobylskij 2020: 412.

###### Type species.

*Aphidiusflavipes* Ratzebrug, 1844, by monotypy.

###### Material examined.

Several species from Palaearctic region were studied. For example:

– *Orthostigmabeyarslani* Fischer, 1995: Spain: • ♀, Alicante, Torrevieja, Natural Park of Lagunas de la Mata-Torrevieja, 25.v.2004 (ENV).

– *Orthostigmalaticeps* (Thompson, 1895): Spain: • ♀, Alicante, Alcoi, Natural Park of Carrascal de La Font Roja, 20.v.2004 (ENV).

– *Orthostigmamaculipes* (Haliday, 1838): Spain: • ♀, Castellon, Pobla de Benifassà, Natural Park of Tinença de Benifassà, 26.ix.2005 (ENV).

– *Orthostigmamandibulare* Tobias, 1962: Russia: Holotype: • ♀, Leningradskaya oblast’. Tolmachevo, border of forest near floodplain of Ostrovenka River, 19.viii.1960, Tobias [leg] (ZISP).

– *Orthostigmapumilum* (Nees, 1834): Spain: • ♀, Castellon, Pobla de Benifassà, Natural Park of Tinença de Benifassà, 17.vi.2004 (ENV).

– *Orthostigmasculpturatum* (Tobias, 1962): Spain: • ♀, Castellon, Pobla de Benifassà, Natural Park of Tinença de Benifassà, 28.viii.2006 (ENV).

###### Diagnosis.

Main characters for the subgenus Orthostigma are the long first flagellar segment (longer than second segment), the notauli only anteriorly present on the mesoscutum and fore wing with vein 2-SR present and more or less distinctly sclerotised.

**Figure 36.**
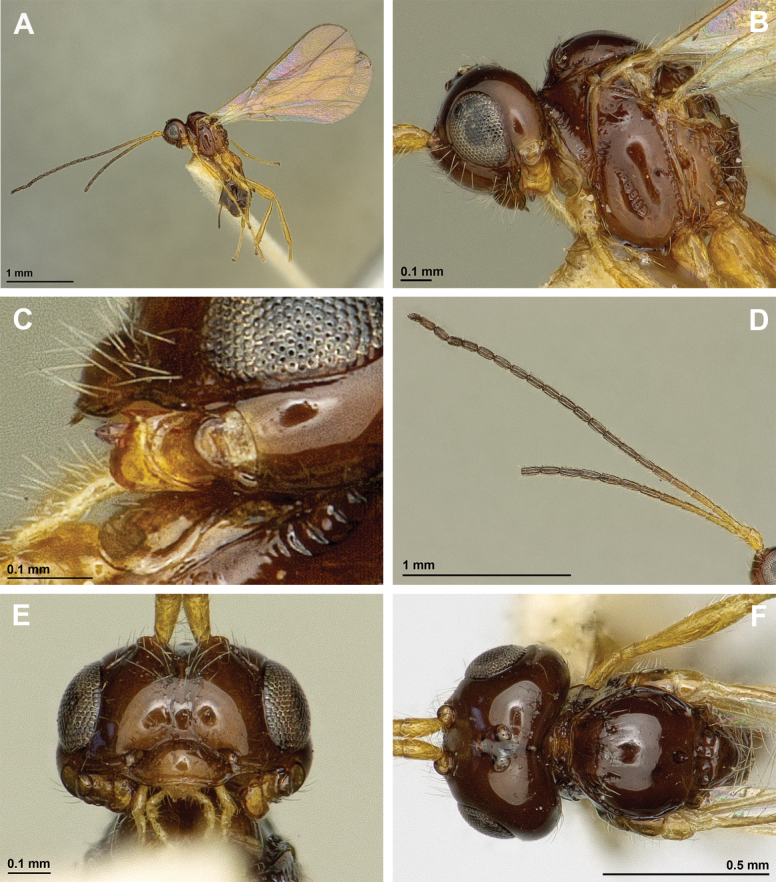
Orthostigma (Orthostigma) mandibulare (Tobias, 1962) (female) **A** habitus, lateral view **B** head and mesosoma, lateral view **C** mandible **D** antenna **E** head, front view **F** head and messonotum, dorsal view.

**Figure 37.**
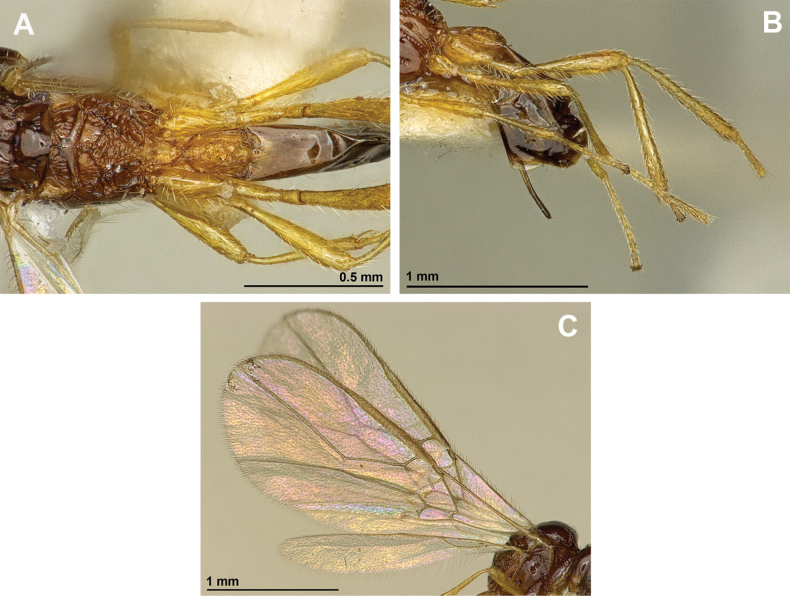
Orthostigma (Orthostigma) mandibulare (Tobias, 1962) (female) **A** propodeum, and metasomal tergites, dorsal view **B** legs, metasoma and ovipositor, lateral view **C** fore and hind wings.

###### Remarks.

This is the largest subgenus with about 60 known species from the Holarctic, Oriental, and Australasian regions.

##### ﻿ Subgenus Patrisaspilota

Taxon classificationAnimaliaHymenopteraBraconidae

Fischer, 1995

B49E3BEC-908B-5DE8-AD9B-DF5F063A61F0

Patrisaspilota Fischer, 1995: 721; Fischer, 2002: 102; 2004: 78; 2010: 636; Wharton 2002: 91 (as subgenus of Orthostigma); Yu et al. 2016; Peris-Felipo et al. 2019: 366; Peris-Felipo and Belokobylskij 2020: 412.

###### Type species.

**Figure 38.**
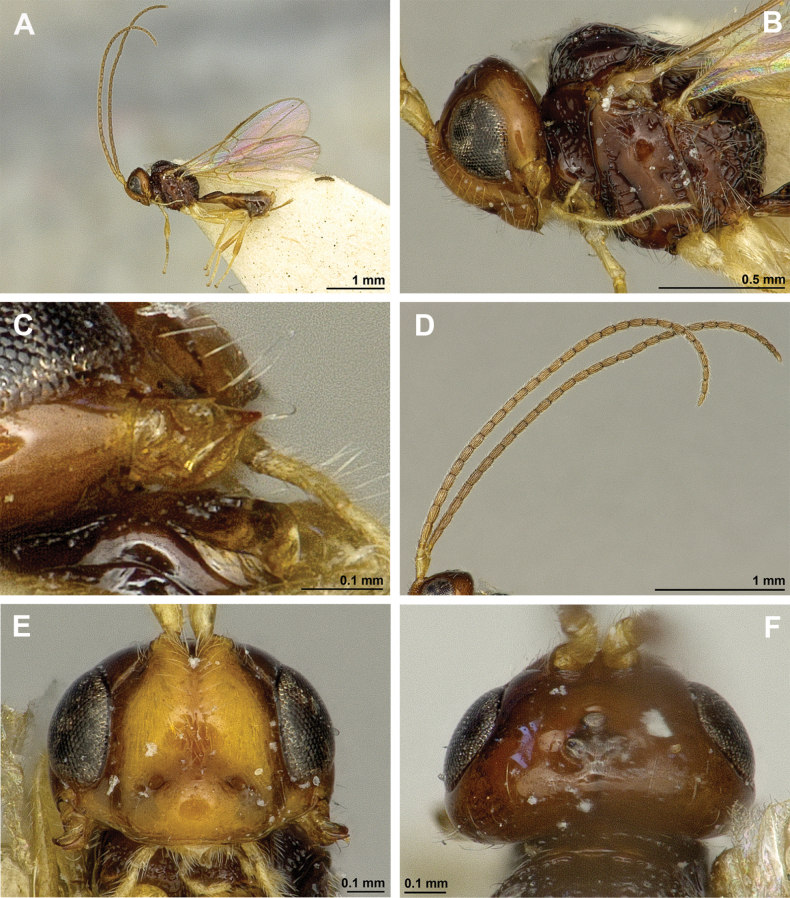
Orthostigma (Patrisaspilota) multicarinatum Tobias, 1990 (holotype, female) **A** habitus, lateral view **B** head and mesosoma, lateral view **C** mandible **D** antenna **E** head, front view **F** head, dorsal view.

**Figure 39.**
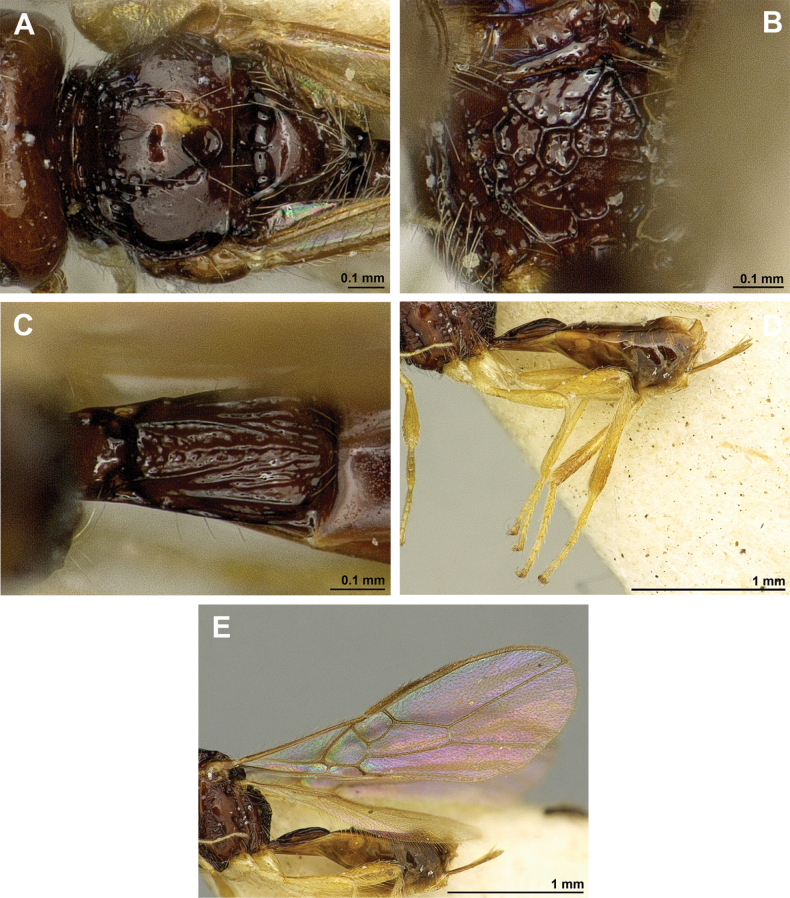
Orthostigma (Patrisaspilota) multicarinatum Tobias, 1990 (holotype, female) **A** mesonotum, dorsal view **B** propodeum **C** first metasomal tergite **D** legs, metasoma and ovipositor, lateral view **E** fore and hind wings.

*Patrisaspilotamemoranda* Fischer, 1995 (= *Orthostigmamulticarinatum* Tobias, 1990) by original designation (Figs 38, 39). Synonymised by Peris-Felipo et al. 2019.

###### Material examined.

***Holotype*** (Orthostigma (Patrisaspilota) multicarinatum) Vietnam: • ♀, Bathuok, 125 km W of Thanh Hoa, prov. Thanh Hoa, 26.i.1989 (B. Korotyaev leg.) (ZISP). ***Paratype*** (Orthostigma (Patrisaspilota) multicarinatum) Vietnam: • ♀, Vietnam, Tram Lap, 20 km N of Buon Luoi, prov. Gia Lai – Con Tum, 6.xii.1988 (Sharkov leg.) (ZISP).

###### Diagnosis.

This subgenus shares the main characters of *Orthostigma* but has the notauli almost reaching the mesoscutal pit.

###### Remarks.

The four Oriental species of this subgenus share the almost completely developed notauli as in Dinotrema (Alitha). The notauli consist of a row of closely located large points more or less reaching the mesoscutal pit. The presence of such type of notauli in different genera of the subtribe Aspilotina (*Orthostigma* and *Dinotrema*) is obviously a parallelism and perhaps indicates the limited value of the character, viz., at least as subgeneric character.

##### ﻿ Subgenus Whartonstigma

Taxon classificationAnimaliaHymenopteraBraconidae

Peris-Felipo, 2020

DA7DCF48-73C4-5492-9833-AE11921FCCFB

Whartonstigma Peris-Felipo in Peris-Felipo & Belokobylskij, 2020: 412 (as subgenus of Orthostigma).

###### Type species.

**Figure 40.**
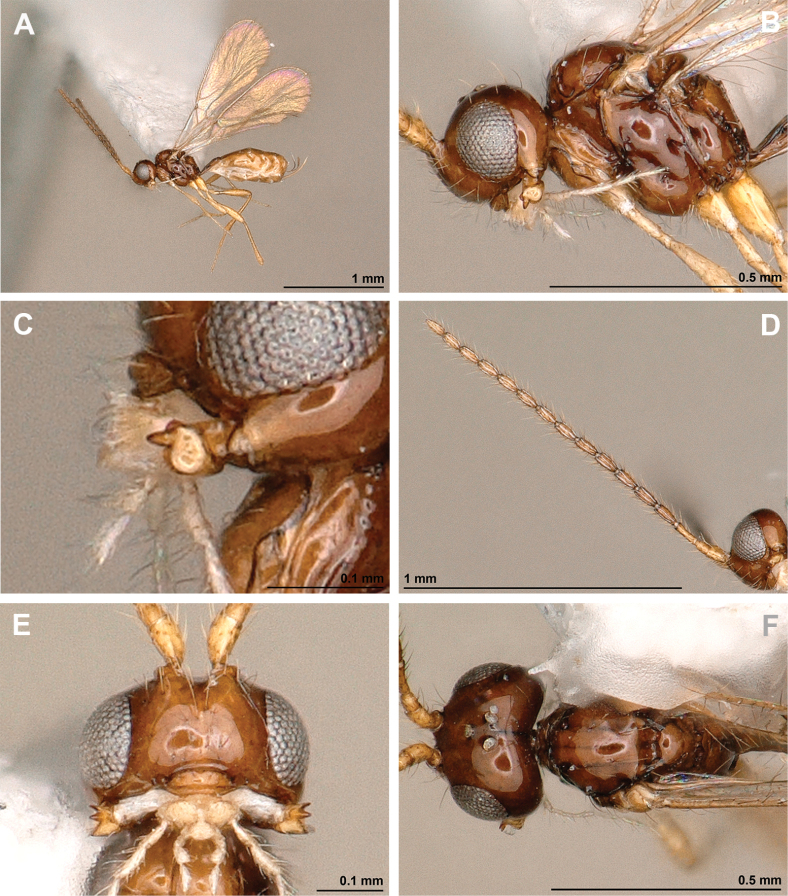
Orthostigma (Whartonstigma) gallowagiWharton 2002 (holotype, female) **A** habitus, lateral view **B** head and mesosoma, lateral view **C** mandible **D** antenna **E** head, front view **F** head and mesonotum, dorsal view.

**Figure 41.**
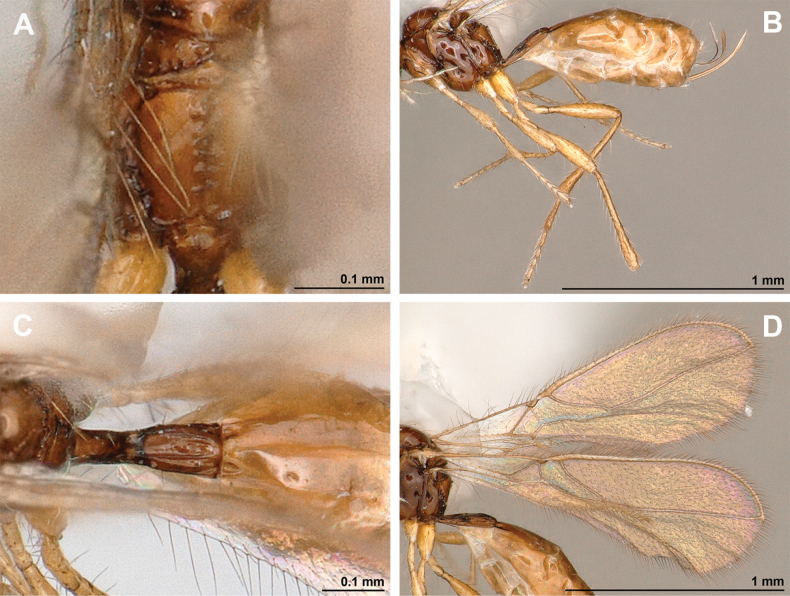
Orthostigma (Whartonstigma) gallowagiWharton 2002 (holotype, female) **A** propodeum **B** legs, metasoma and ovipositor, lateral view **C** first metasomal tergite **D** fore and hind wings.

*Orthostigmagallowagi* Wharton, 2002, by original designation (Figs 40, 41).

###### Material examined.

***Holotype*** (Orthostigma (Whartonstigma) gallowagi): Australia: • ♀, Queensland, Wongabel S. F., 6 km S of Atherton, 12.xi–1.xii.1983, Storey and Brown. M.T. (QMBA). ***Paratypes*** (Orthostigma (Whartonstigma) gallowagi): Australia: • 1 ♀, 1 ♂, same data as holotype [No.111581] (ANIC).

###### Diagnosis.

Very similar to the subgenus Orthostigma sensu stricto but differs from it by the absence of vein 2-SR of the fore wing.

###### Remarks.

This recently described subgenus includes four species from Australia and Papua New Guinea (Peris-Felipo and Belokobylskij 2020).

### ﻿Key to subgenera and genera of the subtribe Aspilotina

**Table:** 

1	First metasomal tergite without dorsope. Second metasomal tergite sculptured medio-basally (except in *A.levis*) (Fig. 42A)	***Apronopa***
–	First metasomal tergite always with dorsope. Second metasomal ter­gite always completely smooth (Fig. 42B)	**2**
2(1)	Mandible with distinct and curved transverse carina. Third tooth usually widest and often lobe-shaped (Fig. 43)	[***Orthostigma* group**] **3**
–	Mandible without curved transverse carina. Third tooth usually narrow and not lobe-shaped (Fig. 44)	[***Aspilota* group**] **8**
3(2)	Vein r of fore wing originating far from base of pterostigma (Fig. 45A). Combined first and second submarginal cells extremely enlarged	***Cubitalostigma***
–	Vein r of fore wing originating near base of pterostigma (Fig. 45B). Combined first and second submarginal cells not enlarged	**3**
4(3)	Paraclypeal fovea large and reaching or almost reaching inner border of eye (Fig. 46A). Furrow between antennal socket and inner margin of eye absent	***Neorthostigma***
–	Paraclypeal fovea smaller, reaching at most halfway distance between clypeus and eye (Fig. 46B). Furrow between antennal socket and inner margin of eye present	[***Orthostigma***] **5**
5(4)	First flagellar segment shorter than second flagellar segment (Fig. 47A)	**Orthostigma (Africostigma)**
–	First flagellar segment either as long as or longer than second flagellar segment (Fig. 47B, C)	**6**
6(5)	Notauli present posteriorly and reaching mesoscutal pit (Fig. 48A) or nearly so	**Orthostigma (Patrisaspilota)**
–	Notauli absent in posterior half of mesoscutum (Fig. 48B)	**7**
7(6)	Vein 2-SR present (Fig. 49A). Vein r distinctly angled with vein 3-SR	**Orthostigma (Orthostigma)**
–	Vein 2-SR absent (Fig. 49B). Vein r + 3-SR gently curved	**Orthostigma (Whartonstigma)**
8(2)	Paraclypeal fovea enlarged and reaching inner border of eye (Fig. 50A, B)	[***Aspilota***] **9**
–	Paraclypeal fovea short, at most halfway distance between clypeus and inner border of eye (Fig. 50C)	**11**
9(8)	Notauli complete and well developed in posterior half of mesoscutum, reaching to mesoscutal pit (Fig. 51A)	**Aspilota (Alitha)**
–	Notauli absent in posterior half of mesoscutum (Fig. 51B, C)	**10**
10(9)	Vein 2-SR of fore wing present (Fig. 52A). Angle between r and 3RSa present and distinct	**Aspilota (Aspilota)**
–	Vein 2-SR of fore wing absent (Fig. 52B). Angle between r and 3RSa absent, resulting in a gently curved or almost straight vein	**Aspilota (Eusynaldis)**
11(8)	Subdiscal cell of fore wing open posteriorly (Fig. 53A). Veins CU1b and 2-1A absent (Fig. 53B)	**12**
–	Subdiscal cell of fore wing completely closed posteriorly (Fig. 53C). Veins CU1b and 2-1A present (Fig. 53D)	**13**
12(11)	Vein 2-SR of fore wing present (Fig. 54A). Precoxal sulcus present (Fig. 54C)	***Lysodinotrema***
–	Vein 2-SR of fore wing absent (Fig. 54B). Precoxal sulcus absent (Fig. 54D)	***Dinostigma***
13(11)	Fore femur with wide and obtuse ventral tooth (Fig. 55A) or with 2 or 3 small teeth	***Leptotrema***
–	Fore femur usual, without ventral teeth (Fig. 55B)	**14**
14(13)	Metasoma with strongly retracted apical sternites under dorsal distally elongated tergites (Fig. 56A)	***Synaldotrema***
–	Metasoma without strongly retracted apical sternites under distal tergites (Fig. 56B)	**15**
15(14)	Scutellum with transverse crenulate depression subposteriorly (Fig. 57A). Wing of female always strongly shortened and with partly thickened veins (Fig. 57B)	***Panerema***
–	Scutellum without transverse crenulate depression subposteriorly (Fig. 57C). Wing of female very rarely shortened and without thickened veins (Fig. 57D)	[***Dinotrema***]. **16**
16(15)	Pterostigma of fore wing narrow, its maximum width less than length of vein r (Fig. 58A)	**17**
–	Pterostigma of fore wing broad (especially in male), its maximum width larger than length of vein r (Fig. 58B)	**18**
17(16)	Vein 2-SR of fore wing present (Fig. 59A). Angle between veins r and 3-SR present and distinct	**Dinotrema (Dinotrema)**
–	Vein 2-SR of fore wing absent (Fig. 59B). Angle between veins r and 3-SR absent and this part of veins connection only gently curved or straight	**Dinotrema (Synaldis)**
18(16)	Vein 2-SR of fore wing present (Fig. 60A). Angle between veins r and 3-SR present and distinct	**Dinotrema (Prosapha)**
–	Vein 2-SR of fore wing absent (Fig. 60B). Angle between veins r and 3-SR absent and combined veins only gently curved or straight	**Dinotrema (Pseudoprosapha)**

**Figure 42.**
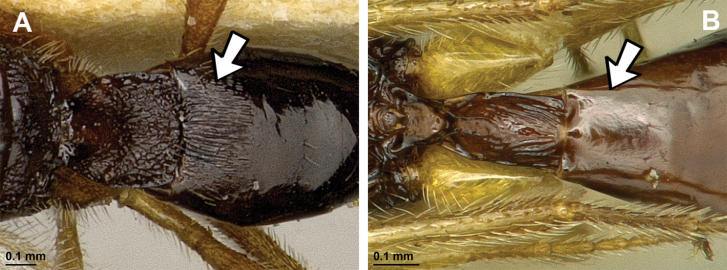
First and second metasomal tergites **A** second metasomal tergite sculptured [*Apronopahaeselbarthi* van Achterberg, 1980] **B** second metasomal tergite smooth [Dinotrema (Dinotrema) katbergense Peris-Felipo, 2016].

**Figure 43.**
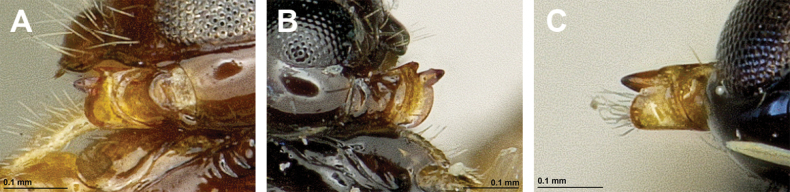
Mandible with distinct and curved transverse carina **A**Orthostigma (Orthostigma) mandibulare (Tobias, 1962) **B**Orthostigma (Africostigma) karkloofense Fischer, 1995 **C***Neorthostigmamacrops* (Stelfox & Graham, 1951).

**Figure 44.**
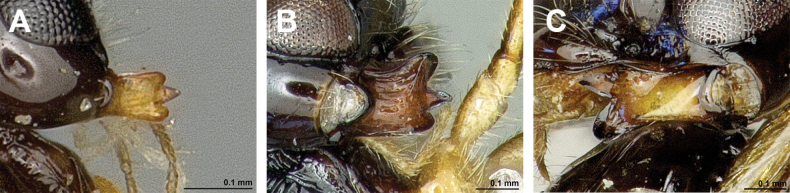
Mandible without curved transverse carina **A**Aspilota (Eusynaldis) varinervis (Zaykov & Fischer, 1972) **B**Dinotrema (Panerema) inops (Foerster, 1863) **C***Grandilotakenyaensis* Fischer, 2002.

**Figure 45.**
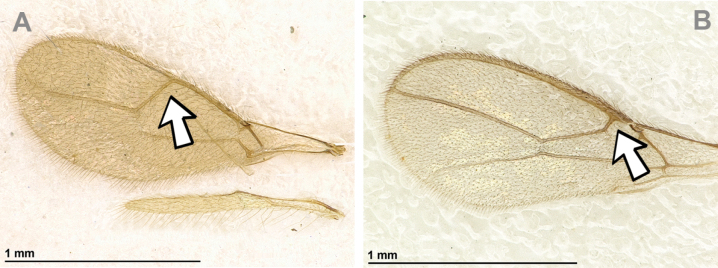
Fore wing **A** vein r originating from middle pterostigma [*Cubitalostigmareichli* Fischer, 1998] **B** vein r originating close to pterostigma base [Dinotrema (Dinotrema) mareum Peris-Felipo, 2013].

**Figure 46.**
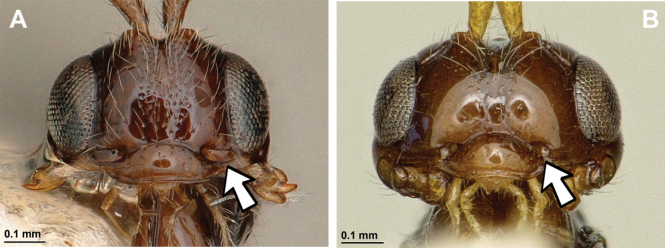
Paraclypeal fovea **A** paraclypeal fovea large and reaching border of eye [*Neorthostigmabrachyclypeata* (Fischer, 1978)] **B** paraclypeal fovea comparatively small [Orthostigma (Orthostigma) mandibulare (Tobias, 1962)].

**Figure 47.**
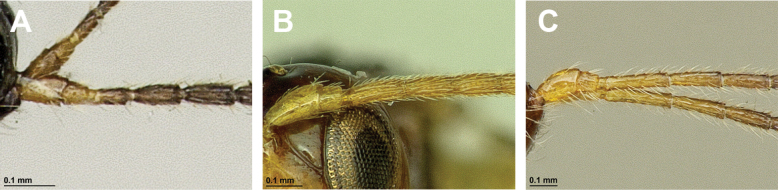
Basal segments of antenna **A**Orthostigma (Africostigma) karkloofense Fischer, 1995 **B**Orthostigma (Patrisaspilota) multicarinatum Tobias 1990 **C**Orthostigma (Orthostigma) mandibulare (Tobias, 1962).

**Figure 48.**
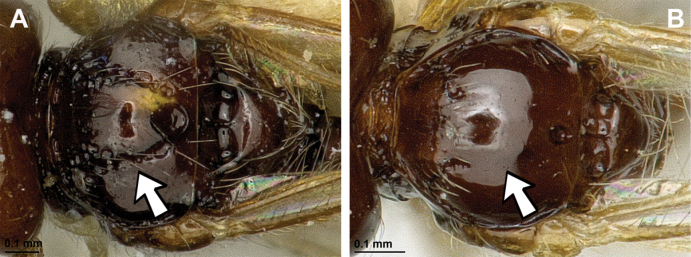
Mesoscutum in dorsal view **A** notauli complete [Orthostigma (Patrisaspilota) multicarinatum Tobias 1990] **B** notauli largely absent posteriorly [Orthostigma (Orthostigma) mandibulare (Tobias, 1962)].

**Figure 49.**
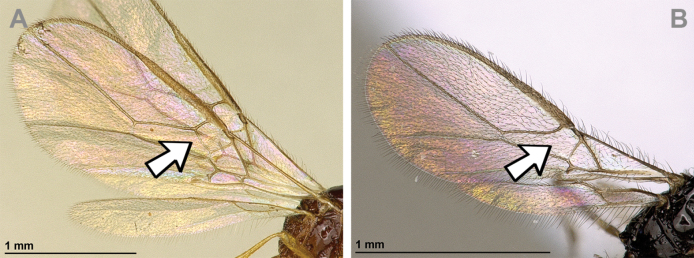
Submarginal cell of fore wing **A** vein 2-SR present [Orthostigma (Orthostigma) mandibulare (Tobias, 1962)] **B** vein 2-SR absent [Orthostigma (Whartonstigma) longipede Peris-Felipo, 2020].

**Figure 50.**
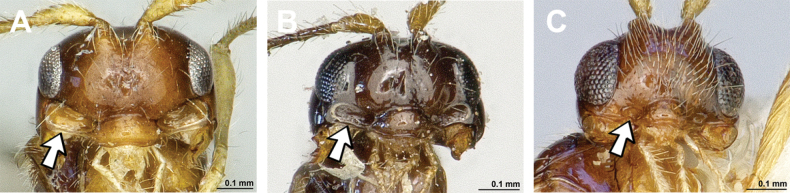
Paraclypeal fovea **A, B** paraclypeal fovea large and reaching border of eye [**A**Aspilota (Aspilota) ajara Peris-Felipo, 2016 **B**Aspilota (Eusynaldis) varinervis (Zaykov & Fischer, 1972)] **C** paraclypeal fovea short [Dinotrema (Dinotrema) multiareolatum Peris-Felipo, 2016].

**Figure 51.**
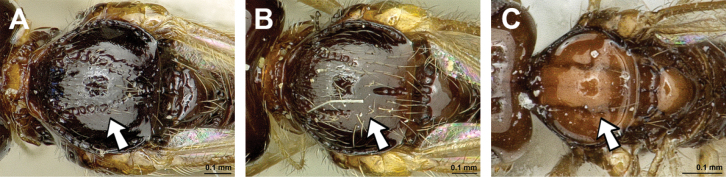
Mesoscutum in dorsal view **A** notauli well developed [Dinotrema (Alitha) vechti (van Achterberg, 1988)] **B, C** notauli incomplete [**B**Dinotrema (Dinotrema) trastoae Peris-Felipo, 2016 **C**Dinotrema (Synaldis) baloghi (Fischer, 1993)].

**Figure 52.**
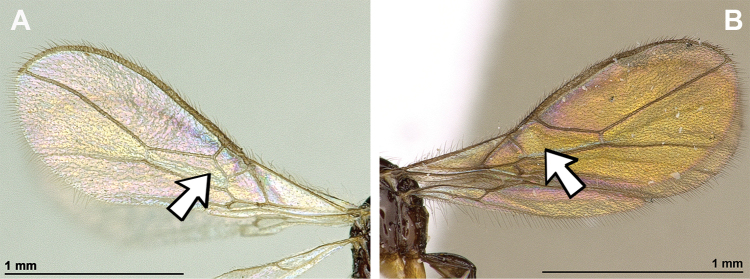
Submarginal cell of fore wing **A** vein 2-SR present [Aspilota (Aspilota) flagimilis Fischer, 1966] **B** vein 2-SR absent [Aspilota (Eusynaldis) villemantae Peris-Felipo, 2019].

**Figure 53.**
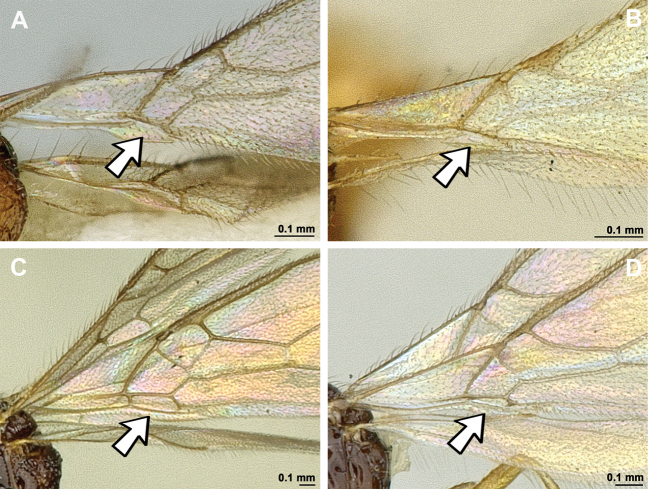
Subdiscal cell **A, B** completely open [**A***Lysodinotremamadli* Fischer, 1995 **B***Dinostigmamuesebecki* Fischer, 1966] **C, D** completely closed [**C**Dinotrema (Dinotrema) trastoae Peris-Felipo, 2016 **D**Dinotrema (Synaldis) longiflagellaris Peris-Felipo, 2017].

**Figure 54.**
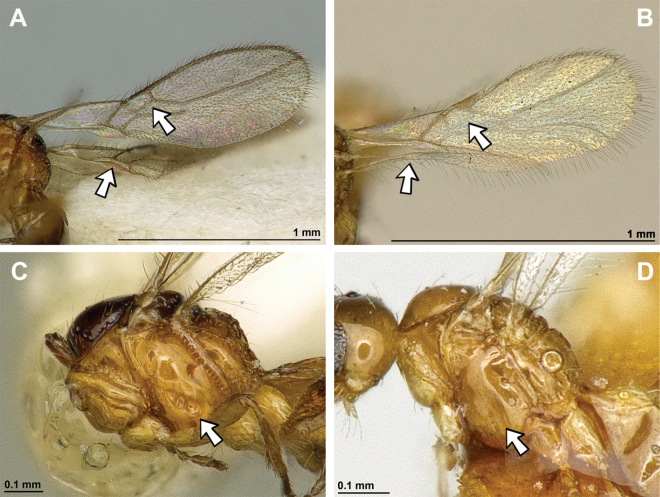
Fore wing and mesosoma **A** vein 2-SR of fore wing present and hind wing with closed cells [*Lysodinotremamadli* Fischer, 1995] **B** vein 2-SR of fore wing absent and hind wing without closed cells [*Dinostigmamuesebecki* Fischer, 1966] **C** precoxal sulcus present [*Lysodinotremamadli* Fischer, 1995] **D** precoxal sulcus absent [*Dinostigmamuesebecki* Fischer, 1966].

**Figure 55.**
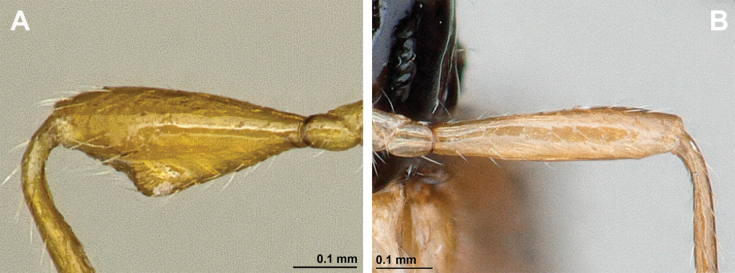
Fore femur **A** fore femur with ventral tooth [*Leptotremadentifemur* (Stelfox, 1943)] **B** fore femur without ventral tooth [Dinotrema (Dinotrema) alysiae Munk & Peris-Felipo, 2013].

**Figure 56.**
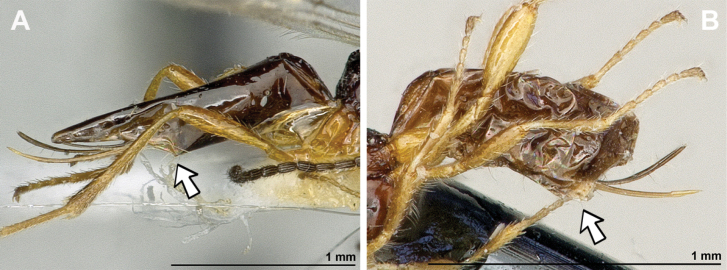
Metasoma, lateral view **A** metasoma with strongly retracted apical sternites [Dinotrema (Synaldotrema) speciosum Belokobylskij & Tobias, 2002] **B** metasoma without retracted sternites [Dinotrema (Synaldis) soederlundi (Fischer, 2003)].

**Figure 57.**
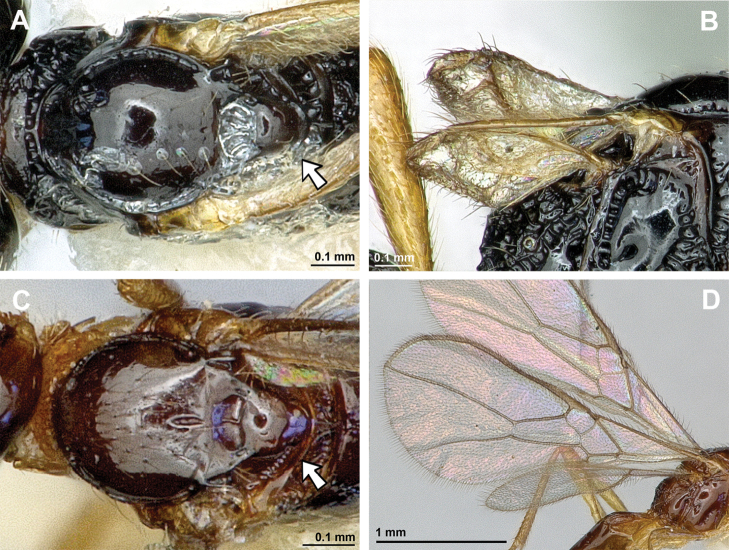
**A** scutellum with crenulate subposterior depression [Dinotrema (Panerema) inops (Foerster, 1863)] **B** shortened wings (female) [idem] **C** scutellum without crenulate [Dinotrema (Dinotrema) multiareolatum Peris-Felipo, 2016] **D** wings (female) depression [idem].

**Figure 58.**
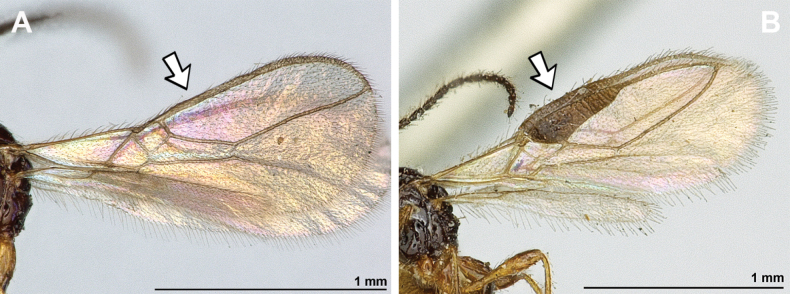
Fore wing **A** pterostigma narrow [Dinotrema (Dinotrema) angusticorne (Fischer, 1969)] **B** pterostigma broad [Dinotrema (Prosapha) speculum (Haliday, 1838)].

**Figure 59.**
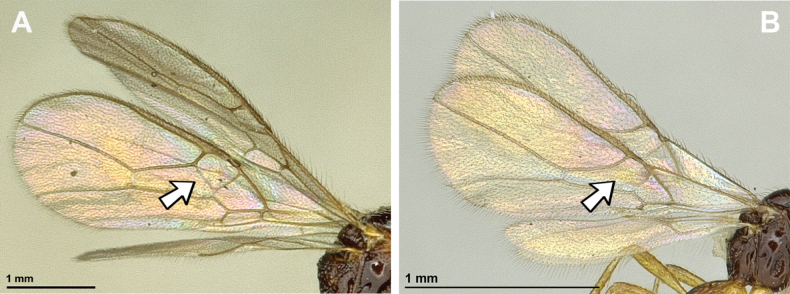
Fore wing **A** vein 2-SR present [Dinotrema (Dinotrema) trastoae Peris-Felipo, 2016] **B** vein 2-SR absent [Dinotrema (Synaldis) longiflagellaris Peris-Felipo, 2017].

**Figure 60.**
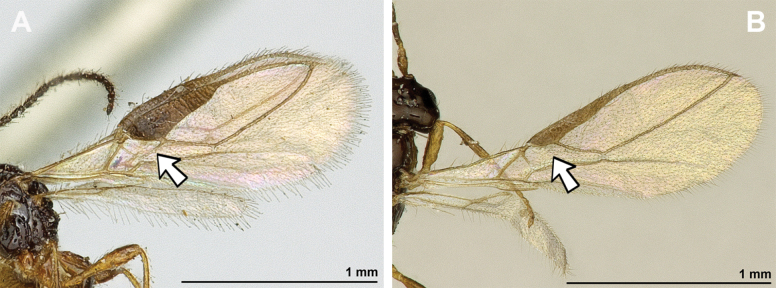
Fore wing **A** vein 2-SR present [Dinotrema (Prosapha) speculum (Haliday, 1838)] **B** vein 2-SR absent [Dinotrema (Pseudoprosapha) stenosoma (van Achterberg, 1988)].

## ﻿Discussion

The alysiine belonging to the subtribe Aspilotina are a relatively homogeneous group of taxa with only a few available diagnostic characters for the determination of its genera and subgenera. This taxonomic group is characterised by several homoplesian features, which are developed parallel in different genera. The most important for identification of the generic diagnostic characters is the state of mandible (with or without a curved transverse carina): it is the most important character to separate *Orthostigma* and *Neorthostigma* from the other alysiine genera. The complete reduction of the vein 2-SR is an appreciable evolutionary event which is connected with the disappearance of the break (angle) between the veins r and 3-SR, and the connection between both veins is only gently curved or almost straight. In most cases it is distinct character state and useful for separation at least of subgenera (Belokobylskij 2002; Peris-Felipo et al. 2014a). The vein r of fore wing is generally situated near the base (in basal quarter) of pterostigma in practically all taxa of this group with only exception the Oriental *Cubitalostigma* where it arises almost from the middle of pterostigma. Besides this, the subdiscal cell of the fore wing is closed by veins 2-1A and CU1b in most of the genera. However, *Lysodinotrema* and *Dinostigma* have this cell open distally through the absence of the veins 2-1A and usually CU1b.

In the hind wing, sclerotised veins usually close the basal and subbasal cells. However, as an exception, there are species with no closed cells in the monotypic Nearctic genus *Dinostigma* (in the modern sense), *Lysodinotrema* and some very derived small species of the genus *Dinotrema*. Besides the wing venation characters, some other valuable features are found on the mesosoma. For example, the notauli are predominantly developed in the anterior subvertical third of the mesoscutum and absent in its posterior horizontal part, except in the subgenera Orthostigma (Patrisaspilota) and Dinotrema (Alitha), where they are complete or nearly so and rather well developed dorso-posteriorly. In addition to these characters, the variation of the propodeal sculpture from entirely smooth or smooth with delineated basolateral areas and often a large areola to entirely finely or coarsely rugose-reticulate and sometimes also with more or less visible delineated areola, shows a considerable intraspecific variation.

Hopefully, the revised classification for the subtribe Aspilotina presented in the current work will facilitate the identification of the genera and subgenera and will allow a better understanding of the character variability in this very complicate and speciose group of genera. Further revisions of this subtribe with use of molecular data will allow for a better insight in each of the genera and subgenera.

## Supplementary Material

XML Treatment for Apronopa

XML Treatment for Aspilota

XML Treatment for Aspilota

XML Treatment for Subgenus Eusynaldis

XML Treatment for Subgenus Grandilota

XML Treatment for Dinostigma

XML Treatment for Dinotrema

XML Treatment for Subgenus Alitha

XML Treatment for Subgenus Dinotrema

XML Treatment for Subgenus Prosapha

XML Treatment for Pseudoprosapha Peris-Felipo, subgen. nov.

XML Treatment for Subgenus Synaldis

XML Treatment for Leptotrema

XML Treatment for Lysodinotrema

XML Treatment for Panerema

XML Treatment for Synaldotrema

XML Treatment for Cubitalostigma

XML Treatment for Neorthostigma

XML Treatment for Orthostigma

XML Treatment for Subgenus Africostigma

XML Treatment for Subgenus Orthostigma

XML Treatment for Subgenus Patrisaspilota

XML Treatment for Subgenus Whartonstigma

## ﻿Additional information

### Author ORCIDs

Francisco Javier Peris-Felipo https://orcid.org/0000-0001-9929-3277

Fernando Santa https://orcid.org/0000-0001-6309-2908

Cornelis van Achterberg https://orcid.org/0000-0002-6495-4853

Sergey A. Belokobylskij https://orcid.org/0000-0002-3646-3459

### Data availability

All of the data that support the findings of this study are available in the main text.

## ﻿Appendix 1

**Table A1.** The distances between genera/subgenera generated from the multivariate statistical approach of the diagnostic characters.

**Group**	**Genus/subgenus**	**Apronopa**	**Aspilota (Aspilota)**	**Aspilota (Eusynaldis)**	**Aspilota (Grandilota)**	**Dinostigma**
**Aspilota**	***Apronopa***	0	1.750	1.800	1.836	2.347
**Aspilota**	**Aspilota (Aspilota)**	1.750	0	0.347	0.416	1.764
**Aspilota**	**Aspilota (Eusynaldis)**	1.800	0.347	0	0.565	1.680
**Aspilota**	**Aspilota (Grandilota)**	1.836	0.416	0.565	0	1.638
**Aspilota**	***Dinostigma***	2.347	1.764	1.68	1.638	0
**Aspilota**	**Dinotrema (Alitha)**	1.470	0.759	0.873	0.917	1.768
**Aspilota**	**Dinotrema (Dinotrema)**	1.642	0.459	0.54	0.667	1.626
**Aspilota**	**Dinotrema (Prosapha)**	1.742	0.729	0.679	0.942	1.425
**Aspilota**	**Dinotrema (Pseudoprosapha)**	1.844	0.917	0.729	1.106	1.389
**Aspilota**	**Dinotrema (Synaldis)**	1.706	0.608	0.459	0.794	1.547
**Aspilota**	***Leptotrema***	1.982	1.211	1.294	1.333	1.987
**Aspilota**	***Lysodinotrema***	1.895	0.994	1.087	0.758	1.194
**Aspilota**	***Panerema***	2.035	1.295	1.122	1.419	1.907
**Aspilota**	***Synaldotrema***	1.982	1.211	1.294	1.333	1.987
**Orthostigma**	***Cubitalostigma***	2.239	1.599	1.472	1.462	1.958
**Orthostigma**	***Neorthostigma***	2.244	1.352	1.398	1.459	2.234
**Orthostigma**	**Orthostigma (Africostigma)**	2.172	1.500	1.557	1.595	2.179
**Orthostigma**	**Orthostigma (Orthostigma)**	1.879	1.033	1.074	1.167	1.856
**Orthostigma**	**Orthostigma (Patrisaspilota)**	1.762	1.242	1.317	1.367	2.009
**Orthostigma**	**Orthostigma (Whartonstigma)**	1.933	1.105	1.033	1.242	1.785
**Group**	**Genus/subgenus**	**Dinotrema (Alitha)**	**Dinotrema (Dinotrema)**	**Dinotrema (Prosapha)**	**Dinotrema (Pseudoprosapha)**	**Dinotrema (Synaldis)**
**Aspilota**	***Apronopa***	1.470	1.642	1.742	1.844	1.706
**Aspilota**	**Aspilota (Aspilota)**	0.759	0.459	0.729	0.917	0.608
**Aspilota**	**Aspilota (Eusynaldis)**	0.873	0.540	0.679	0.729	0.459
**Aspilota**	**Aspilota (Grandilota)**	0.917	0.667	0.942	1.106	0.794
**Aspilota**	***Dinostigma***	1.768	1.626	1.425	1.389	1.547
**Aspilota**	**Dinotrema (Alitha)**	0	0.483	0.747	0.967	0.678
**Aspilota**	**Dinotrema (Dinotrema)**	0.483	0	0.502	0.723	0.347
**Aspilota**	**Dinotrema (Prosapha)**	0.747	0.502	0	0.347	0.47
**Aspilota**	**Dinotrema (Pseudoprosapha)**	0.967	0.723	0.347	0	0.502
**Aspilota**	**Dinotrema (Synaldis)**	0.678	0.347	0.470	0.502	0
**Aspilota**	***Leptotrema***	1.218	1.021	1.173	1.331	1.135
**Aspilota**	***Lysodinotrema***	0.998	0.878	1.082	1.247	1.002
**Aspilota**	***Panerema***	1.313	1.140	1.331	1.243	0.96
**Aspilota**	***Synaldotrema***	1.218	1.021	1.173	1.331	1.135
**Orthostigma**	***Cubitalostigma***	1.63	1.484	1.617	1.553	1.36
**Orthostigma**	***Neorthostigma***	1.646	1.501	1.63	1.724	1.555
**Orthostigma**	**Orthostigma (Africostigma)**	1.546	1.37	1.497	1.614	1.446
**Orthostigma**	**Orthostigma (Orthostigma)**	0.909	0.839	1.017	1.145	0.911
**Orthostigma**	**Orthostigma (Patrisaspilota)**	0.839	1.024	1.205	1.354	1.131
**Orthostigma**	**Orthostigma (Whartonstigma)**	1.023	0.905	0.999	1.017	0.839
**Group**	**Genus/subgenus**	**Leptotrema**	**Lysodinotrema**	**Panerema**	**Synaldotrema**	**Cubitalostigma**
**Aspilota**	***Apronopa***	1.982	1.895	2.035	1.982	2.239
**Aspilota**	**Aspilota (Aspilota)**	1.211	0.994	1.295	1.211	1.599
**Aspilota**	**Aspilota (Eusynaldis)**	1.294	1.087	1.122	1.294	1.472
**Aspilota**	**Aspilota (Grandilota)**	1.333	0.758	1.419	1.333	1.462
**Aspilota**	***Dinostigma***	1.987	1.194	1.907	1.987	1.958
**Aspilota**	**Dinotrema (Alitha)**	1.218	0.998	1.313	1.218	1.630
**Aspilota**	**Dinotrema (Dinotrema)**	1.021	0.878	1.140	1.021	1.484
**Aspilota**	**Dinotrema (Prosapha)**	1.173	1.082	1.331	1.173	1.617
**Aspilota**	**Dinotrema (Pseudoprosapha)**	1.331	1.247	1.243	1.331	1.553
**Aspilota**	**Dinotrema (Synaldis)**	1.135	1.002	0.960	1.135	1.360
**Aspilota**	***Leptotrema***	0	1.378	1.592	1.529	1.848
**Aspilota**	***Lysodinotrema***	1.378	0	1.465	1.378	1.528
**Aspilota**	***Panerema***	1.592	1.465	0	1.592	1.790
**Aspilota**	***Synaldotrema***	1.529	1.378	1.592	0	1.848
**Orthostigma**	***Cubitalostigma***	1.848	1.528	1.790	1.848	0
**Orthostigma**	***Neorthostigma***	1.85	1.773	1.926	1.850	1.885
**Orthostigma**	**Orthostigma (Africostigma)**	1.771	1.648	1.836	1.771	1.793
**Orthostigma**	**Orthostigma (Orthostigma)**	1.365	1.249	1.445	1.365	1.367
**Orthostigma**	**Orthostigma (Patrisaspilota)**	1.553	1.376	1.620	1.553	1.561
**Orthostigma**	**Orthostigma (Whartonstigma)**	1.450	1.337	1.306	1.450	1.230
**Group**	**Genus/subgenus**	**Neorthostigma**	**Orthostigma (Africostigma)**	**Orthostigma (Orthostigma)**	**Orthostigma (Patrisaspilota)**	**Orthostigma (Whartonstigma)**
**Aspilota**	***Apronopa***	2.244	2.172	1.879	1.762	1.933
**Aspilota**	**Aspilota (Aspilota)**	1.352	1.5	1.033	1.242	1.105
**Aspilota**	**Aspilota (Eusynaldis)**	1.398	1.557	1.074	1.317	1.033
**Aspilota**	**Aspilota (Grandilota)**	1.459	1.595	1.167	1.367	1.242
**Aspilota**	***Dinostigma***	2.234	2.179	1.856	2.009	1.785
**Aspilota**	**Dinotrema (Alitha)**	1.646	1.546	0.909	0.839	1.023
**Aspilota**	**Dinotrema (Dinotrema)**	1.501	1.37	0.839	1.024	0.905
**Aspilota**	**Dinotrema (Prosapha)**	1.63	1.497	1.017	1.205	0.999
**Aspilota**	**Dinotrema (Pseudoprosapha)**	1.724	1.614	1.145	1.354	1.017
**Aspilota**	**Dinotrema (Synaldis)**	1.555	1.446	0.911	1.131	0.839
**Aspilota**	***Leptotrema***	1.85	1.771	1.365	1.553	1.45
**Aspilota**	***Lysodinotrema***	1.773	1.648	1.249	1.376	1.337
**Aspilota**	***Panerema***	1.926	1.836	1.445	1.62	1.306
**Aspilota**	***Synaldotrema***	1.85	1.771	1.365	1.553	1.45
**Orthostigma**	***Cubitalostigma***	1.885	1.793	1.367	1.561	1.23
**Orthostigma**	***Neorthostigma***	0	1.63	1.126	1.354	1.194
**Orthostigma**	**Orthostigma (Africostigma)**	1.63	0	0.972	1.252	1.074
**Orthostigma**	**Orthostigma (Orthostigma)**	1.126	0.972	0	0.483	0.347
**Orthostigma**	**Orthostigma (Patrisaspilota)**	1.354	1.252	0.483	0	0.678
**Orthostigma**	**Orthostigma (Whartonstigma)**	1.194	1.074	0.347	0.678	0
